# Mismatch and G-Stack Modulated Probe Signals on SNP Microarrays

**DOI:** 10.1371/journal.pone.0007862

**Published:** 2009-11-17

**Authors:** Hans Binder, Mario Fasold, Torsten Glomb

**Affiliations:** Interdisciplinary Centre for Bioinformatics, Universität Leipzig, Leipzig, Germany; University of Southampton, United Kingdom

## Abstract

**Background:**

Single nucleotide polymorphism (SNP) arrays are important tools widely used for genotyping and copy number estimation. This technology utilizes the specific affinity of fragmented DNA for binding to surface-attached oligonucleotide DNA probes. We analyze the variability of the probe signals of Affymetrix GeneChip SNP arrays as a function of the probe sequence to identify relevant sequence motifs which potentially cause systematic biases of genotyping and copy number estimates.

**Methodology/Principal Findings:**

The probe design of GeneChip SNP arrays enables us to disentangle different sources of intensity modulations such as the number of mismatches per duplex, matched and mismatched base pairings including nearest and next-nearest neighbors and their position along the probe sequence. The effect of probe sequence was estimated in terms of triple-motifs with central matches and mismatches which include all 256 combinations of possible base pairings. The probe/target interactions on the chip can be decomposed into nearest neighbor contributions which correlate well with free energy terms of DNA/DNA-interactions in solution. The effect of mismatches is about twice as large as that of canonical pairings. Runs of guanines (G) and the particular type of mismatched pairings formed in cross-allelic probe/target duplexes constitute sources of systematic biases of the probe signals with consequences for genotyping and copy number estimates. The poly-G effect seems to be related to the crowded arrangement of probes which facilitates complex formation of neighboring probes with at minimum three adjacent G's in their sequence.

**Conclusions:**

The applied method of “triple-averaging” represents a model-free approach to estimate the mean intensity contributions of different sequence motifs which can be applied in calibration algorithms to correct signal values for sequence effects. Rules for appropriate sequence corrections are suggested.

## Introduction

Genomic alterations are believed to be the major underlying cause of common diseases such as cancer [1]. These alterations include various types of mutations, translocations, and copy number variations. Single nucleotide polymorphisms (SNPs) are the most abundant type of polymorphism in the human genome. With the parallel developments of dense SNP marker maps and technologies for high-throughput SNP genotyping, SNPs have become the polymorphic genetic markers of choice for genetic association studies which aim at discovering the genetic background of different phenotypes. Microarray platforms are capable of parallel genotyping of hundreds of thousands of SNPs in one measurement. To date this high throughput technology is therefore routinely performed to get comprehensive genome wide information about the genetic variability of individuals in genome wide association studies.

The microarray technology utilizes the specific affinity of fragmented DNA to form duplexes with surface-attached oligonucleotide probes of complementary sequence and subsequent optical detection of bound fragments using fluorescent markers. The measured raw probe intensities are subject to large variability, and depend not only on the abundance of allelic target sequences, but also on other factors such as the sequence dependent probe binding affinity. The successful correction of raw probe signals for such parasitic effects is essential to obtain exact genotyping estimates. It requires identification and understanding of the main sources of signal variation on the arrays.

The main purpose of this paper is to analyze the variability of probe signals of Affymetrix GeneChip SNP arrays as a function of the probe sequence and to identify relevant sequence motifs which significantly modulate the probe signals. Such sequence motifs constitute potential building blocks for improved calibration methods which aim at correcting probe signals for sequence effects.

The discovery of characteristic sequence motifs using SNP arrays is also important in a more general context: DNA/DNA duplex formation is the basic molecular mechanism of functioning not only of SNP arrays but also of other array types such as re-sequencing [2] and different expression arrays (gene- or exon-related and whole genome tiling arrays) of newer generations. It has been demonstrated that thermodynamic models of hybridization taking into account such sequence-dependent effects are capable to significantly reduce signal fluctuation between probes interrogating the same target [3]–[6]. Knowledge of the underlying physical process is however still lacking in many details despite the recent progress in this field (see, for example, [7]–[12]). Particularly, surface hybridization is different from oligonucleotide duplexing in solution (see e.g. [13]–[15]). Systematic studies on oligonucleotide interactions on microrrays are therefore required to tackle selected problems such as signal anomalies of poly-guanine runs [16], [17], the specific effect of mismatched base pairings [4], [15], [18] and/or the positional dependence of interaction strengths [9], [19].

The presented analysis takes special advantage of the probe design used on GeneChip SNP arrays. Particularly, this technology uses 25meric oligonucleotide probes corresponding to a perfect match for each of the two allele sequences. In addition, a mismatch probe is synthesized for each allele to detect non-specific binding. Combination of this information with the target composition of fractionated genomic DNA used for hybrization on the arrays enables us to deduce the base pairings in the probe/target complexes producing a particular probe intensity. Making use of the hundreds of thousands signal values per SNP array allows us to extract specific intensity contributions of selected short sequence motifs of two-to-four adjacent nucleotides via appropriate averaging. The obtained motif-specific intensity contributions characterize the stability of the involved base pairings which include all relevant combinations of canonical Watson-Crick and mismatched pairings. Finally, the systematic analysis of different sequence motifs such as triples of adjacent bases allows us to identify those which account for significant signal variations.

We previously performed an analogous chip study using intensity data of expression arrays to characterize base pair interactions in DNA/RNA hybrid duplexes [20] which in final consequence enabled us to develop an improved algorithm for signal calibration and quality control [5], [6]. Note that, compared with expression arrays, SNP arrays are even better suited to study base pair interactions because probe/target-duplexes are typically less contaminated with non-specific target fragments of unknown sequence and because genomic copy numbers are less variable than mRNA-transcript concentrations.

The paper is laid out as follows: Section 2 sets out the method and, particularly, explains the classification criteria used to assign the probe intensities to different interaction modes. In Section 3, we analyze different factors which affect the probe intensities such as the number of mismatches, the optical and non-specific background, signal contributions due to different sequence motifs such as different base triples, single and tandem mismatches and their positional dependence along the sequence. In addition we assess symmetry relations of the motifs, their decomposition into nearest neighbor terms and compare the results with thermodynamic nearest neighbor parameters characterizing DNA/DNA interactions in solution. In Section 4 we discuss the stability of different mismatches and discover the possible origin of the “poly-G” effect. Finally, we suggest rules for selecting appropriate sequence motif to adequately correct the probe signals for sequence effects which might serve as the basic ingredient of improved calibration methods.

## Methods

### Probe design for SNP detection

SNP arrays intend to determine genotype and copy numbers of hundreds of thousands of bi-allelic single nucleotide polymorphism- (SNP) loci in one measurement. Let us specify each SNP by the alternative nucleotides in the sense DNA-strand of allele A and allele B using the convention B_A_/B_B_, where B_A_/B_B_∈{A/C, A/G, A/T, C/G, C/T, G/T} stands for one of six SNP types considered on GeneChip SNP microarrays. These SNP types are either complementary (cSNP: A/T, C/G) for substitutions of complementary nucleotides or non-complementary (ncSNP) otherwise.

**Figure 1:**
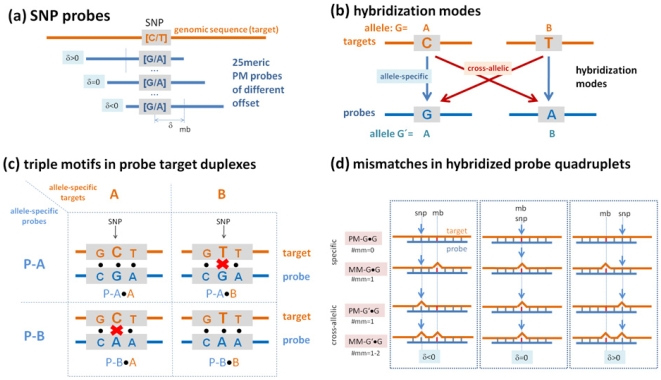
Probe design and hybridization modes for SNP detection. (a) Each SNP (for example [C/A]) is probed by 25meric probes of complementary sequence. Different offsets δ of the SNP position relative to the middle base (mb) of the probe sequence are used. In addition, each PM probe is paired with one MM probe the middle base of which mismatches the target sequence (not shown). (b) The allele-specific probes intend to detect the respective targets via allele-specific binding which however competes with cross-allelic hybridization of targets of the alternative allele (see also the reaction equation Eq. (6)). (c) Both hybridization modes give rise to four different types of probe/target duplexes formed by the two allele-specific probes. The figure shows the respective base pairings for a selected SNP-triple which consists of the SNP [C/T] and its nearest neighbors. Mismatched non-canonical pairings are indicated by crosses. (d) Each box includes one probe-quartet which consists of two PM/MM-probe pairs interrogating either targets of allele G = A or targets of allele G' = B and vice versa (i.e. G = B and G' = A). Only targets of one allele are assumed to be present as in the sample. They hybridize to the probes of both allele sets forming either specific or cross-allelic duplexes, respectively. The three selected probe quartets differ in the offset δ of the SNP position (see arrows and part a of the figure) relatively to the middle base of the probe. The different combinations give rise to different numbers and positions of mismatched pairings which are indicated by the bulges. Their number varies between #mm = 0 and #mm = 2 in dependence on the probe type, hybridization mode and offset position. Complete probe-sets use 10 probe quartets.

On Affymetrix 100k GeneChips, each allele is interrogated by ten perfect match (PM)-probes, the 25meric sequence of which perfectly matches the genomic target-sequence at the selected SNP position (see Figure 1 for illustration). The probes differ in their SNP position which is shifted by different offsets relative to the middle base, δ∈{−4,…,0,…,+4}. Between three and seven of the PM probes refer to the sense strand and the remaining seven to three probes refer to the antisense strand.

Each PM-probe is paired with one mismatch (MM)-probe of identical sequence except the middle base which intends to estimate the contribution of non-specific background hybridization to the respective PM-probe intensity. Note that the mismatched pairing noticeably reduces specific binding of the respective target to the MM probes compared with the respective PM-probe. The middle base is substituted by its Watson-Crick complement as standard (for example A↔T) except for the probes interrogating cSNPs with offset δ = 0, i.e. in the middle of the probe sequences. The non-complementary replacements A↔G and T↔C are realized in this special case to avoid inter-allelic specific binding to the MM (see below).

Taken together, each allele of each SNP is probed by a set of 20 PM/MM probe pairs. These, in total 40 probe split into two sub-sets of 10 probe pairs for each allele which we will term ‘allele-set’. Each allele-set consists of probes with the SNP interrogation position placed at the sense and antisense strands and moving the 25meric probe sequence up and down the target sequence with respect to the SNP locus by different offsets to improve the accuracy of genotyping and copy number estimates.

Both allele sets use the same offset positions. Therefore each particular offset, δ, is probed by one probe pair for each allele. These four probes (i.e. two PM/MM-pairs) addressing each offset position make up the so-called probe-quartet referring to the same 25-meric segment of the target genome (see Figure 1).

### Hybridization modes on SNP arrays

SNP microarrays are hybridized with fragmented genomic DNA representing the targets for the probes attached on the chip surface. Let us consider one SNP locus of a heterozygous genotype: The hybridization solution of genomic DNA consequently contains targets of both alleles A and B. The hybridization reactions can be described by three coupled equations for each probe,
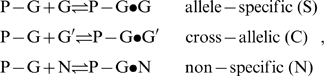
(1)where P-G (P = PM, MM) denotes the probes which are designed to interrogate targets of allele G = A, B. G' = B, A are the targets of the respective alternative allele.

In the allele-specific hybridization mode (called S-mode) the probes bind the target which they intend to detect via duplex formation of the type P-A•A and P-B•B, respectively. In the cross-allelic hybridization mode (C-mode) the probes bind targets of the alternative allele in duplexes of the type P-A•B and P-B•A, respectively. The considered probes also bind non-specific genomic fragments not referring to the selected SNP. Such non-specific duplexes are of the type P-A•N and P-B•N where N subsumes all non-specific target sequences with non-zero affinity to the selected probe.

In the S-mode the PM probes completely match the target sequence whereas in the C-mode the PM-sequence mismatches the target at the SNP position. The respective MM probes mismatch the target either only at the middle position (S-mode) or at both the middle and the SNP position (C-mode). The respective base pairings are specified below.

The measured intensity of each probe represents the superposition of contributions originating from the three hybridization modes, and from the optical background caused by the dark signal of the scanner and by residual fluorescent markers not attached to target-fragments,
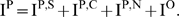
(2)In a first order approximation, the intensity-contributions are directly related to the respective number of probe/target-duplexes (indicated by the square brackets),
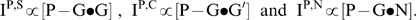
(3)The non-specific and optical background contributions used in Eq. (2) are, on the average, independent of the probe type (e.g., I^PM,N^≈I^MM,N^). We combine both contributions into one mean background intensity
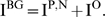
(4)Its fraction and the fraction of non-specific hybridization,
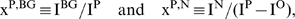
(5)define the percentage of background intensity in the total signal and the percentage of non-specific hybridization signal in the total signal after correction for the optical background, respectively.

### Homozygous-present and homozygous–absent probes

Three types of targets compete for duplex formation with each probe in the general case considered in Eq. (1). In the special case of homozygous genotypes only targets of one allele are present in the hybridization solution. As a consequence, the types of competing targets per probe reduce to two ones, namely non-specific and either allele-specific or cross-allelic targets. Particularly, the probes targeting the present allele hybridize specifically (homozygous-present probes) whereas the probes interrogating the alternative allele hybridize in the cross-allelic mode (homozygous-absent probes), i.e.
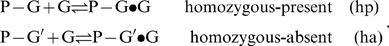
(6)Eq. (2) applies to the special situations of homozygous-present and -absent hybridizations with I^P,C^ = 0 and I^P,S^ = 0, respectively (see Figure 1 for illustration).

### Matched and mismatched base pairings in probe/target duplexes

In this section we specify the base pairings formed in the probe/target duplexes at two selected sequence positions, namely that of the SNP- and that of the middle-base of the probe sequence. The SNP position is shifted by the offset δ with respect to the middle base. SNP- and middle-base are consequently identical for δ = 0.

In the specific hybridization mode the PM probes perfectly match the respective target-allele forming Watson-Crick (WC) pairings along the whole probe sequence including the two selected positions (Figure 1 and Text S1). Contrarily, one mismatched pairing occurs at the SNP position of the PM probe upon cross-allelic hybridization. The MM probe always forms a mismatched pairing at the middle position and, upon C-hybridization, also at the SNP position. For δ≠0 the MM-duplexes contain consequently two mismatches with the special case δ = ±1 referring to so-called tandem-mismatches of two adjacent mismatched pairings. For |δ|>1 the two mismatches are separated by at least one WC pairing. The MM form only one mismatch in the C-hybridization mode for δ = 0 because the mismatched SNP position equals the middle base.

The assignment of the specific and cross-allelic hybridization modes to the six probed bi-allelic SNP types B_A_/B_B_ (see above) and the two probe types (P = PM, MM) provides the full set of 16 possible base pairings in the probe/target duplexes at their SNP- and/or middle-position (see Text S1). We classify the pairings into canonical Watson-Crick pairs (referred to as At-group; upper and lower case letter refer to the probe and target sequences, respectively), and three groups of mismatches (Aa-, Ag- and Ac-group). The notations of the groups are chosen in agreement with the respective pairing formed by an adenine in the probe sequence (see Text S1 for the details). The mismatched groups refer to self-complementary pairings (Aa-group: Aa, Tt, Gg, Cc), to self-paired (Ag-group: Ag, Tc, Ga, Ct) and cross-paired (Ac-group: Ac, Tg, Gt, Ca) pyrimidines and purines, respectively. Note that these groups are invariant with respect to the strand direction because complementary substitutions do not change the group membership.

The number and the type of the mismatches are not specified in probe/target duplexes formed in the non-specific hybridization mode. Nevertheless, the sequence effect can be described in terms of the properties of canonical WC pairings [20], [21]. This result seems to contradict the fact that non-specific duplexes are per definition destabilized at minimum by one, but typically by more mismatched pairings. Note however, that these mismatch-effects are averaged out by calculating mean binding characteristics of WC-interactions (At-group) which stabilize the non-specific duplexes.

### Interaction modes

As discussed in the previous subsections, the probe/target duplexes are characterized by the hybridization mode (h = S, C, N) and a series of probe attributes: probe-type (P = PM, MM), probe sequence and middle base (B_13_ = A,T,G,C), strand direction (d = s, as), SNP type (B_A_/B_B_), and SNP offset (δ = −4,…,0,…,+4). Each particular combination of the hybridization mode with a set of probe attributes unambiguously determines the interaction mode between probe and target. It is characterized by

- the base pairing at the SNP position and at the middle position, which includes all 16 pairwise combinations of nucleotides, 4 of which form WC pairings and 12 of which are mismatches;
- Watson-Crick pairings at the remaining positions of the probe sequence;
- the mutual shift between the middle and the SNP base by up to four bases in both directions (δ);
- different numbers of mismatches per duplex varying between #mm = 0 (for P = PM and h = S) and #mm = 2 (P = MM and h = C, only δ≠0);
- different relative positions of paired mismatches (#mm = 2) which are either separated by at least two WC pairings (|δ|>1) or form tandem-mismatches (|δ| = 1).

The design of SNP GeneChips thus enables us to study how these interaction modes affect the probe intensities in a systematic way. Vice versa, the probe intensities are related to the amount of bound DNA-targets which, in turn, depends on the stability of the duplexes and thus on the binding constant of the respective interaction mode. Knowledge of the binding constant and of the interaction mode then allows us to compute the genotype call and copy number of a given SNP.

### SNP array data

Intensity-data of the 100k GeneChip SNP array and supplementary files were downloaded from suppliers website (https://www.affymetrix.com/support/technical/sample_data/hapmap_trio_data.affx). This data set was specially designed for the development and evaluation of low-level analysis methods for genotyping and copy number estimation from probe intensity data (see, e.g., [22]). Particularly we analyzed array NA06985_Xba_B5_4000090 taken from the Mapping 100k HapMap Trio Dataset (100K_trios.xba.1.zip) including library- and annotation information (probe sequences, fragment lengths and GC-content of the targets, GCOS-genotype calls). We use the genotypes provided by Affymetrix for the array data and select only homozygous SNP loci for further analysis (41,629 homozygous out of 58,960 total loci, ∼70.1%). In this special case the hybridization mode is either specific or cross-allelic for homozygous-present and homozygous-absent alleles, respectively (see Eq. (6)).

The data are further filtered to remove probe intensities which are dominated by nonspecific hybridization by more than x^P,N^>0.2 (Eq. (5)). These selection criteria are chosen from the hook plot of the chip data which is briefly described in the supporting text (see Text S1 and also refs. [5], [6]). This special type of analysis characterizes the hybridization quality of each chip. Interestingly, the data obtained reveal that nonspecific hybridization contributes to the signal intensities of SNP arrays to a smaller degree compared with expression arrays in agreement with previous results [9]. This difference can be rationalized by the smaller heterogeneity of genomic DNA copies (with respect to their sequences and fragment-lengths) and especially by the smaller range of copy number variations compared with the range of variation of mRNA-transcript concentrations. The latter values can cover several orders of magnitude whereas the former ones typically change by a factor of less than ten.

The intensity data are corrected for the optical background intensity and for residual non specific hybridization before further analysis as described in Text S1.

### Triple averaged intensities and probe sensitivities

We previously used the so-called ‘triple-averaging’ approach to estimate the effective strength of base pairings in probe/target duplexes on GeneChip expression arrays [20]. This approach analyzes the effect of the sequence on the probe intensities using triples of neighboring bases. It accounts for the fact that the strength of a selected base pair interaction in oligonucleotide duplexes is significantly modulated by the two adjacent pairings on both sides of the selected base.

Let us define the standard triple as the string of three consecutive bases (xBy) in 5′→3′-direction of the probe sequence (x,B,y∈A,T,G,C) where the nearest neighbors (x, y) of the central base B form Watson-Crick pairs in the duplexes with the targets. The position of the triples along the probe sequence was chosen in such a way that its central base (B) agrees either with the middle base (mb) or with the SNP base (see Figure 1c for illustration). The triple is consequently centered about the middle base of the probe (δ = 0) or shifted by δ sequence positions up or downwards (δ≠0). The hybridization mode and the probe attributes unambiguously define the base pairing of the center base, Bb (b∈a,t,g,c), according to the selected interaction group, Ab = At, Aa, Ag or Ac (see Text S1). The Ab-group can be chosen by applying appropriate criteria of probe selection.

So-called triple averages of the intensity are calculated as log-mean over all probes within the classes defined by the interaction group of the central base (Ab = At, Aa, Ag or Ac), by the triple motif xBy at offset position (δ = −4,…,0,…, +4) and by the number of mismatches per duplex (#mm = 0, 1 or 2)
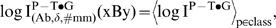
(7)with T = G,G' for hp- and ha-probes, respectively. A series of nested mean values can be generated by averaging over one or more of the attributes given by class = (Ab, δ, #mm). For example, 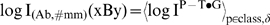 denotes averaging over the offset positions δ and 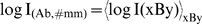 refers in addition to averaging over the triple motifs xBy to get the mean intensity per interaction group.

The triple sensitivities are defined as the deviation of the triple-averaged intensity from an appropriately chosen mean value over all triples (see below and [23]), e.g.,
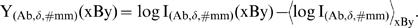
(8)It is reasonable to assume that the strand direction does not affect the strength of the respective base pairings. In our analyses we therefore pool the probes which are assigned to the same interaction mode independently of their strand direction (d = s, as) assuming that the respective genotypes are properly assigned on both strands.

### Tandem and flanking mismatches

Special selection criteria for triples with one flanking mismatch and of tandem mismatches are given in the scheme shown in Text S1. The former motif is characterized by the usual standard triple as defined in the previous section which is however flanked on one side by a mismatched pairing, i.e. w(xBy)m (w∈At; m∈Aa,Ag,Ac). Tandem mismatches are two adjacent mismatches present in homozygous-absent duplexes of the MM-probes with SNP offset positions |δ| = 1. Both motifs were separately analyzed to estimate the specific effect of flanking and of tandem mismatches in comparison with the standard triples.

## Results

### SNP offset position and the number of mismatches

**Figure 2:**
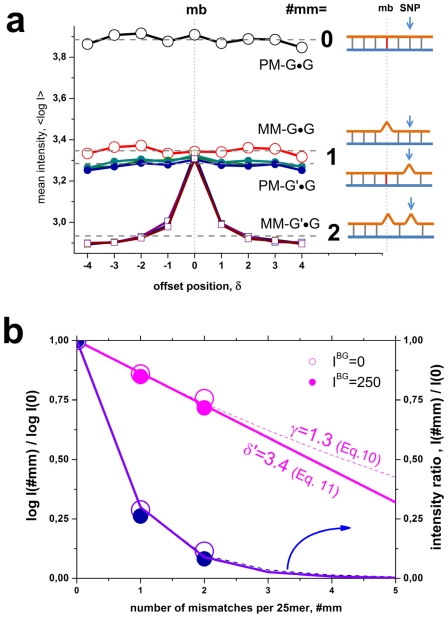
SNP offset and number of mismatches. Averaged log-intensities for probes of different mismatch-groups and offset-positions, (panel a) and mean effect of the number of mismatches (#mm) on the observed intensity (panel b). Panel a: Mean probe intensity (averaged over all probes with a given SNP offset, see the arrow in the schematic drawing in the right part for illustration) as a function of the offset-position of the mismatch with respect to the middle base (δ) for different number of mismatches per probe/target duplex (#mm = 0…2). Virtually no significant effect of the offset-position was observed for single mismatches within the relevant range |δ|<5. Contrarily, the mean intensity decreases with increasing separation between double mismatches (#mm = 2) where one is located in the centre of the probe (middle base, mb) and the second one at offset position δ. Note that both mismatches merge into one for δ = 0. The homozygous-absent data (P-G'•G) were separately calculated for the three groups of mismatches, Aa, Ac and Ag: The respective curves are almost identical. Panel b: Relative decrease of the mean probe intensity as a function of #mm (symbols). The curves are calculated using Eqs. (9) and (10). The data are shown in logarithmic (left axis, upper data) and linear (right axis) scale without (open symbols) and with (solid symbols) background correction.

The specific and cross-allelic hybridization modes include perfect matched and mismatched probe/target duplexes with up to two mismatched pairings at the SNP- and/or mb-position (see Text S1). To study the effect of the number of mismatched pairings, #mm, and the effect of the SNP offset position, δ, on the intensities we calculate the log-intensity averages, 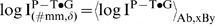, for each SNP offset of homozygous-present (T = G) and absent probes (T = G', see part a of Figure 2 and Eq. (7)).

The SNP base of each probe forms a WC pairing in P-G•G duplexes (hp-mode). The respective averaged intensities per SNP position are consequently pseudo-replicates of different sub-ensembles of probes referring to the same interaction mode, namely perfectly-matched (PM-G•G) or single-mismatched (MM-G•G) probe/target duplexes (see the schematic drawings in panel a of Figure 2). The scattering of the respective data about their mean thus reflects the variability of the obtained intensity averages in the different sub-ensembles of probes.

In P-G'•G duplexes (allele absent/ha-mode) the SNP base forms a mismatched pairing. The averaged intensities consequently refer to the shift of the mismatch relative to the middle base. For the PM probes (PM-G'•G) the position of the respective single mismatch only weakly affects the mean intensity in the relevant range of SNP offsets (panel a of Figure 2). This result is in agreement with previous studies which show that the destabilizing effect of single mismatches is almost constant over a broad range in the middle part of short-length oligonucleotide duplexes and decreases only for the last 4–6 base positions near the ends of the probe sequence [24]–[26].

In contrast, the MM-probes form two mismatches in the homozygous-absent mode (MM-G'•G) at the SNP- (for |δ|>0) and at the middle position. Both mismatches are separated by (δ−1) WC pairings in-between. The observed mean intensity decreases with increasing distance between the mismatches (panel a of Figure 2). This trend indicates that the destabilizing effect of the mismatches is small for neighboring tandem mismatches (|δ| = 1); it slightly increases for a single intermediate WC pairing (|δ| = 2) and it essentially levels off for more WC pairings in between (|δ|>2).

The presented results show that the number of mismatched pairings per duplex (#mm) is the most relevant factor which affects the mean intensity of the probes (see the horizontal lines in Figure 2, panel a). The logarithmic-intensity ratio can be approximated as function of #mm by [27]

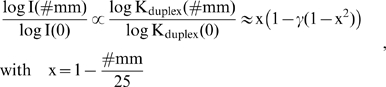
(9)where 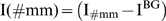 is the background corrected intensity of probes with #mm mismatches; K_duplex_(#mm) denotes the respective mean association constant of probe/target duplexes with #mm mismatches; x is the fraction of WC pairings in the duplex and γ is a fit-constant depending on the hybridization conditions.

An alternative, simple “mismatch”-function results from the assumption of additive contributions of each base pairing, 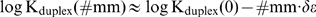, where log K_duplex_ is related to the free energy of duplex stability and δε is its mean incremental penalty (in units of logK_duplex_) if one substitutes one WC pairing by a mismatch. This approach predicts an exponential decay of the intensity as a function of the number of mismatches, 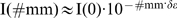, which transforms into
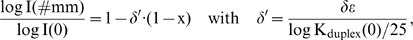
(10)using the logarithmic form as in Eq. (9). The constant δ′ is given by the ratio between the incremental penalty due to the mismatch and logK_duplex_(0)/25, which has the meaning of a mean additive contribution of one WC pairing to log K_duplex_(0). Panel b of Figure 2 shows that both alternative functions given by Eqs. (9) and (10) are virtually not distinguishable for #mm<3. They can be used to extrapolate the intensity values to #mm>2 in a rough approximation. The data show that one and two mismatches reduce the intensity to about 25% and 10% of its initial value, respectively. Eqs. (9) and (10) predict that more than two mismatches decay the intensit to tiny values of less than 5% of its value for perfect matched duplexes. The estimated value of the decay rate δ′>3 in Eq. (10) indicates that the intensity penalty due to the first two mismatches markedly exceeds the average intensity contribution of a single WC pairing in the perfect matched probe/target duplexes. Simple balance considerations imply that δ′ has to decrease with increasing number of mismatches as predicted by Eq. (9) (see also the theoretical curves in part b of Figure 2).

### Positional dependence of single base- and triple-motifs

**Figure 3:**
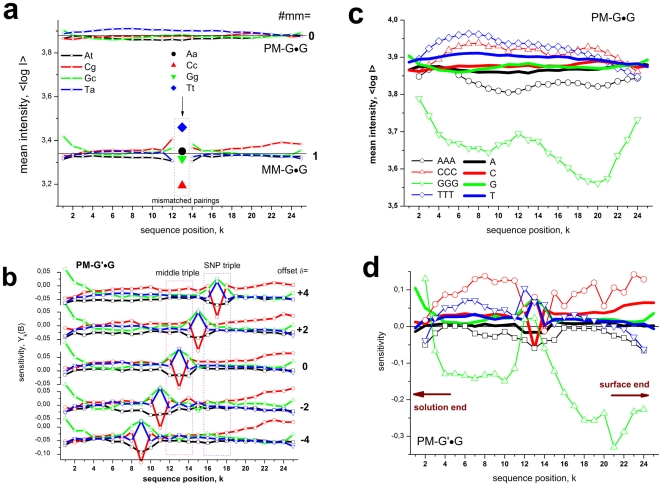
Positional dependence of the probe intensities. Panel a: Single base data of allele-specific (S-mode) PM and MM probes. Each data point was calculated as log-intensity average over all probes of the considered class with the indicated base at position k of the probe sequence. It is associated either with WC pairings or with mismatched pairings at the middle base (mb)-position of the MM. These mismatches give rise to markedly larger variability of the intensities than the WC pairings do at the remaining positions. Panel b shows the positional dependence of the sensitivity (deviation of the log-intensity from its mean over all probes of the class) of cross-allelic PM probes (C-mode) with different offsets of the SNP. The base at the SNP position forms a mismatched pairing which shifts along the sequence according to the offset. Note that the mismatch-values are averages over all groups (Aa, Ag, Ac; see Text S1) whereas the mismatches in part a of the figure refer to the Aa-group. Panel c enlarges the single-base curves for PM-G•G shown in panel a. In addition, mean log-intensity values were calculated for homo-triples along the probe sequence (the position k refers to the center base of the triples). The mean log-intensities slightly increase for AAA, CCC and TTT compared with the single-base averages but markedly decrease for triple guanines. Panel d shows the respective single-base and triple values for the cross-allelic PM data for offset δ = 0 shown in panel b. Comparison with panel c indicates subtle differences of the curves at positions which refer to WC pairings in both situations: For example, triple-guanines motifs give rise to relatively large intensities near the surface end of the probe and also the cytosines (C- and especially CCC-motifs) are associated with largest intensities for most of the WC pairings in part d whereas thymines give rise to largest intensities in part c.

The PM probes form exclusively WC pairings in homozygous-present PM-G•G duplexes. We calculated log-mean intensities for all these duplexes containing a certain base (B = A,T,G,C) at each position k = 1…25 of the probe sequence to study the positional effect of WC-base pairings over the whole sequence length (see lines in panel a of Figure 3). The obtained positional-dependent log-intensity averages only weakly vary about their total mean. The base-specific differences essentially disappear towards the right end of the probes (k>23) which is attached to the chip surface (see also panel c of Figure 3).

Also the homozygous-present duplexes of the MM-probes, MM-G•G, form predominantly WC pairings except the middle base which forms mismatches of the Aa-interaction group. The single base averaged intensities of these mismatches vary to a much larger degree about their mean compared to the WC pairings (see the arrow in panel a of Figure 3). The strong mismatch effect extends also to the flanking bases at adjacent positions k = 12 and 14.

Panel b of Figure 3 shows the single-base positional dependence of homozygous-absent PM probes (PM-G'•G) for different offsets δ of the SNP which forms a mismatched pairing in the probe/target duplexes. As for the MM, the SNP position exhibits a larger spread of the single-base values about their mean compared with the WC pairings at the remaining sequence positions. They represent averages over mismatches of the Aa-, Ag- and Ac-type in contrast to the Aa-type mismatches of the middle base shown in panel a of Figure 3. The data clearly reflect the shift of the mismatched pairing with changing offset position of the SNP. The profiles remain nearly invariant at the remaining sequence positions.

To estimate the effect of longer sequence motifs we calculated intensity-averages of probes possessing “homo”-triples, i.e. runs of three consecutive bases of the same type at a certain sequence position (see panel c and d of Figure 3). The specific effect of these motifs clearly exceeds that of the single bases, especially for runs of triple G: These GGG-motifs systematically reduce the probe intensities by a factor of ∼10^−0.2^−10^−0.4^≈0.6−0.4 compared with the mean intensity for most of the sequence positions. In contrast, the mean effect of a single G is almost negligible. The GGG-effect essentially disappears at the mismatch position in the middle of the probe sequence (see panel d of Figure 3 which shows profiles of PM-G'•G probes with δ = 0). The similar “buckled” shape of the GGG-profile in the middle of the probe sequence of PM-G•G duplexes (panel c) probably indicates a certain small fraction of incorrectly assigned genotypes in the selected sub-ensemble of homozygous present probes.

Comparison of panel c and d of Figure 3 reveals also more subtle differences between the profiles at positions which refer to WC pairings in both, the PM-G•G (panel c) and PM-G'•G (panel d) duplexes: Firstly, triple TTT provide the largest intensities for the former duplexes whereas triple CCC become largest in PM-G'•G duplexes. Moreover, the effect of cytosines progressively increases towards the surface end in PM-G'•G duplexes whereas it apparently disappears in the data obtained from PM-G•G duplexes. Secondly, the intensity effect due to guanines begins with positive values at the solution end of PM-G'•G duplexes (k = 1) and then steeply decreases to negative values.

It is known that the sequence profiles are sensitive to factors such as the optical background correction and saturation [18], [28] (see also below). Large and small intensities are prone to saturation and background effects, respectively, which differently affect the specific signal. Saturation, for example, limits large probe intensities and therefore reduces the relative effect of strong-base pairings because probes containing such motifs are most affected by saturation. The relative small single- and triple- cytosine values in the profiles of PM-G•G duplexes can be attributed to selectively stronger saturation of probes containing these motifs. Contrarily, in the PM-G'•G duplexes saturation is much less relevant owing to the smaller average level of probe occupancy and intensity. The different response of triple guanines and cytosines near the solution and surface ends of the probe seems puzzling and will be addressed in the discussion section.

### Triple sensitivities

In the next step we neglect the positional dependence of probe intensities and address the sequence-specific effect of base pairings in triple motifs centered about the middle and SNP base of the probes.

**Figure 4:**
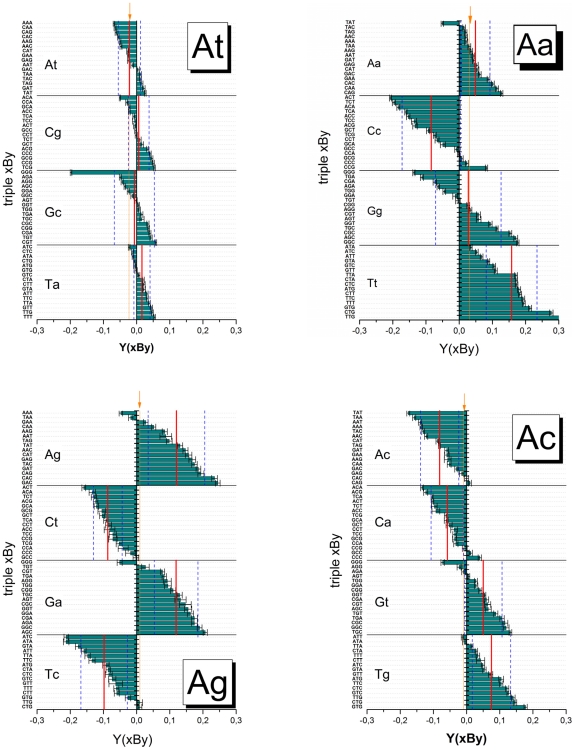
Triple averaged sensitivities. The triple values are calculated using (Eq. (8)) and ranked with increasing sensitivity for each center base B forming matched (group At) and different mismatched (groups Aa, Ag and Ac) pairings with the target as indicated in the figure by upper (probe) and lower (target) letters. The sensitivity-values are calculated relative to the total log-average of all single-mismatched probes of the chip. Sub-averages of the interaction groups (see arrows) and of the central base pairings are shown by vertical solid lines. The vertical dashed lines indicate the standard deviation of the triple values about the central-base related mean (see also Table 1). The mean and the standard deviation estimate the stability of the respective pairing Bb and the effect of flanking WC pairings, respectively. The error bars indicate the standard error of the triple sensitivities.

The triple averaged and background corrected intensities were used to calculate the 64 triple-sensitivity values for each of the four interaction groups, (Eq. (8)). Particularly, we selected the homozygous-absent PM probes (PM-G'•G) with one mismatched pairing at SNP position and used the base-triples centered about the middle base (At-group) and about the SNP base (Aa, Ag, Ac group, see Text S1). All intensities of probes with offset-positions |δ|>1 were log-averaged. The sensitivity values were related to the total mean of all used PM-G'•G probes irrespective of the particular interaction group, i.e.,
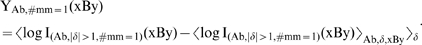
(11)
Figure 4 summarizes the obtained sensitivity data which provide a measure of the specific effects of the pairing of the central base and of their nearest neighbors in terms of the deviation from the mean over the respective group of probes.

Most of the sensitivities of the At-group (WC pairings) relatively tightly scatter about their mean indicating an only moderate sequence effect. The ‘GGG’-triple however strongly deviates from this rule; it causes a relatively large intensity penalty: One ‘GGG’-motif give rise to the reduction of the intensity on the average by a factor of about 10^−0.2^∼0.63 compared with the mean intensity. The triples considered refer to offset positions |δ|≤4 about the middle base. The full positional dependence of ‘GGG’ (Figure 3, part d) actually indicates a stronger intensity drop for sequence positions halfway to the ends. Importantly, the ‘GGG’-penalty is in contradiction to complementary rules because the complementary ‘CCC’-motif reveals completely different sensitivity-properties: Triple C's gives rise to the opposite effect; i.e. they amplify the intensity by a factor of about 10^+0.1^∼1.25. We will discuss this puzzling result below.

The substitution of the central WC pairing by mismatches considerably increases the variability of the triple data. The mean variability of each interaction group was estimated in terms of the standard deviation of all 64 combinations of each group (Table 1): Its value more than doubles for the mismatched groups (SD = 0.09–0.13) compared with the WC-group (SD = 0.04). Single mismatches can modify the intensity by a factor between ∼10^−0.25^ = 0.55 and ∼10^+0.25^ = 1.8. This result generalizes the trend which is illustrated in Figure 3a for the special case of mismatches of the Aa-group in the middle of the probe sequence.

**Table 1:** Sources of variability of triple motifs and of tandem mismatches.

Interaction group^a^	At	Aa	Ag	Ac
Base pairings (Watson Crick or mismatches)	WC pairings: At, Cg, Gc, Ta	self complementary mismatches: Aa, Cc, Gg, Tt	self paired mismatches: Ag, Ct, Ga, Tc	cross paired mismatches: Ac, Ca, Gt, Tg
**triples** b	0.04±0.001	0.12±0.0005	0.13±0.001	0.09±0.0005
**3′/5′-asymmetry** c	0.03	0.11	0.07	0.05
**complementary asymmetry (without GGG)** d	0.05 (0.02)	0.10 (0.08)	0.08 (0.06)	0.06 (0.05)
**NN-residual** e	0.02 (0.02)	0.02 (0.02)	0.03 (0.02)	0.01 (0.01)
**flanking mismatches** f		0.04	0.03	0.02
**tandem mismatches (xy)** g		0.02 (0.033)	0.015 (0.047)	0.013 (0.044)
**tandem mismatches (BB')** g		0.06 (0.07)	0.06 (0.10)	0.05 (0.08)
**tandem mismatches (yB'/B'y)** g		0.05	0.08 (0.055)	0.05

a variability estimates are separately calculated as standard deviation for each Ab-interaction group: SD = √<Δ^2^>_Ab_.

b variability of the triple averages with respect to the group-mean: Δ = Y_Ab_(xBy)−<Y_Ab_(xBy)>_Ab_; it estimates the variability of interactions due to the choice of the triple; the standard error refers to the variability of the probe level data of each interaction group.

c variability of the triple averages after 3′/5′-transformation: Δ = Y_Ab_(xBy)−Y_Ab_(yBx).

d variability of the triple averages after complementary-transformation: Δ = Y_Ab_(xBy)−Y_Ab_(x^c^B^r^y^c^); the values in the brackets are obtained after omitting the GGG-motif.

e variability of the residual values after reduction of the model rank NNN→NN: Δ = Δ^res^_Ab_ (see Eq. (17)).

f variability due to flanking mismatches: Δ = Δ^flank^_Ab_ (see Eq. (15)).

g variability due to quadruplet motifs with tandem mismatches (xBB'y)/(yB'Bx) with B∈Aa and B'∈Aa,Ag,Ac. The SD were calculated with respect to the average over the three groups (Δ(xy) = <Y_Ab_(xBB'y)>_BB'_−<<Y_Ab_(xBB'y)>_BB'_>_Ab_ and Δ(BB') = <Y_Ab_(xBB'y)>_xy_−<<Y_Ab_(xBB'y)>_xy_>_Ab_) and with respect to the total mean over all couples (values in the brackets; (Δ(xy) = <Y_Ab_(xBB'y)>_BB'_−<<Y_Ab_(xBB'y)>_BB'_>_Ab,xy_ and Δ(BB') = <Y_Ab_(xBB'y)>_xy_−<<Y_Ab_(xBB'y)>_xy_>_Ab,BB'_).

### Mean mismatch stability

The mean sensitivity over all triples with a given middle base B provides a measure of the average stability of the respective mismatched pairing Bb (see the red lines in Figure 4). For the Aa-, Ag- and Ac-groups one gets the relations Cc<Gg≈Aa<Tt, Tc≈Ct<Ag≈Ga and Ac≈Ca<Gt≈Tg, respectively. They confirm the expected symmetries for bond reversals Bb→B^r^b^r^ in symmetrical DNA/DNA interactions, i.e. Y_Ab_(Bb)≈Y_Ab_(B^r^b^r^) (for example for Tc→Ct and Ac→Ca). Note that, in contrast, DNA/RNA interactions are asymmetrical in solution [29] and on microarrays [18], [20].

Comparison of the mean sensitivity values for each central pairing of all three mismatch-groups provides the following ranking of the stability of mismatched pairings:
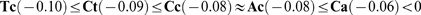
(12)

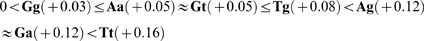
The numbers in the brackets are the respective mean sensitivities for each mismatched pairing averaged over the 16 combinations of adjacent bases (standard error: ∼±0.02).

Other authors report similar rankings of the stability of single-mismatches in DNA/DNA-oligomer duplexes which are obtained from hybridization studies on surfaces (microarrays or special solid supports) or in solution:
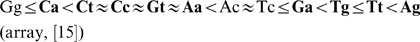


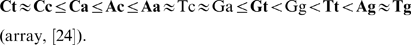
(13)

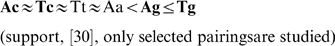


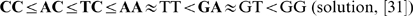
In solution, both dimerized oligonucleotides are equivalent as indicated by the two capital letters which assign the pairing.

Basic agreement of the reference studies with our ranking is highlighted using bold letters. Accordingly, the consensus-ordering of the array-studies comprises Ct, Ca, Cc as low stability mismatches; Ag, Tg, Tt as high stability mismatches and Gt and Aa at the intermediate position. A major difference between the previous rankings occurs for Gg which is the least stable in the study of Naiser et al. [15] and one of the most stable mismatches in the study of Wick et al. [24]. Our data plead for intermediate stability. Inspection of Figure 4 reveals the large variability of triples with a central Gg-mismatch about zero. Imbalanced triple selection in studies using a limited number of oligonucleotides therefore are prone to lead to biased results where the apparent Gg-stability can vary between large and low values in dependence on the particular realization of probe/target-duplexes containing a Gg-mismatch. The total probe number of the studied SNP array (10^6^) exceeds the probe number used in previous studies by about three orders of magnitude (10^3^ [24] and 2–3×10^3^ [15]). Comparison of the different rankings of mismatch strength obtained from chip and solution data reveals disagreement especially for GG, GT and TT motifs. These differences possibly indicate additional or alternative explanations for the inconsistent chip rankings which will be discussed below.

Note also that the reported references [15], [24] estimated mismatch-stabilities by directly comparing the intensities of MM and PM probes, which refers to the stability difference between the mismatched pairing and the respective WC pairing. Our ranking uses the mean stability of all considered single-base mismatches as reference level which is independent of the particular triple. The relatively small variability of the single-base averages of the At-group (see the red lines for the At-group in Figure 4) however show that the explicit use of the WC-sensitivity as reference essentially does not change the ranking of mismatch-stabilities in our data set. Direct comparison with the reference data is therefore adequate within the error limits.

### Symmetries

The triple sensitivities shown in Figure 4 can be examined with respect to two simple symmetry-relations, namely 3′/5′-reversal and probe/target-complementarity,
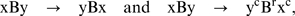
(14)respectively (sequence motifs are ordered in 5′-3′ direction). The superscripts “c” and “r” denote complementary nucleotide letters in the special case of WC pairings (e.g., A^c^ = T) and bond-reversals for the more general situation which includes also mismatched pairings (e.g. A^r^ = G and A^r^ = A for mismatches of the Ag and Aa groups, respectively).

Perfect 3′/5′-symmetry of the triple sensitivities (i.e. Y(xBy) = Y(yBx)) is expected if the base pairings are independent of their nearest neighbors. Stacking interactions between adjacent nucleotides however make an essential contribution to the stability of DNA/DNA-duplexes [32], [33]. The change of stacking contributions after strand-reversal is governed by the different stereochemistry of 3′/5′ and 5′/3′ strand directions in the duplexes. The deviation from the perfect 3′/5′-symmetry relation thus estimates the effect of stacking interactions in the considered triplets.

In contrast, the complementarity relation keeps the strand direction unchanged. Perfect complementarity of the triple sensitivities (i.e. Y(xBy) = Y(y^c^B^r^x^c^)) is expected if both interacting strands are physically equivalent and if their reactivity is not selectively perturbed by parasitic reactions such as intramolecular folding and/or bulk dimerization [13]. For example, duplexing experiments in solution typically use oligonucleotides of equal length and of low propensity for intramolecular folding and self-interactions. A very different situation occurs on microarrays because the reacting partners are highly asymmetric in length and conformational freedom: Firstly, the probes are attached to the chip surface whereas the targets are dissolved in the supernatant solution with consequences for their reactivity. For example, the interactions depend on the position of the nucleotide letter in the probe sequence owing to their attachment to the chip surface which gives rise to positional dependent constraints of probe/target interactions [9], [13]. Secondly, the length of the targets exceeds that of the probes typically by more than one order of magnitude which markedly enhances their propensity for intramolecular folding and intermolecular duplexing reactions in solution in a sequence-dependent fashion with consequences for their effective interactions with the probes. Hence, deviations from perfect complementarity are expected to detect imbalanced probe/target interactions due to the asymmetric nature of the hybridization reaction on microarrays.

**Figure 5:**
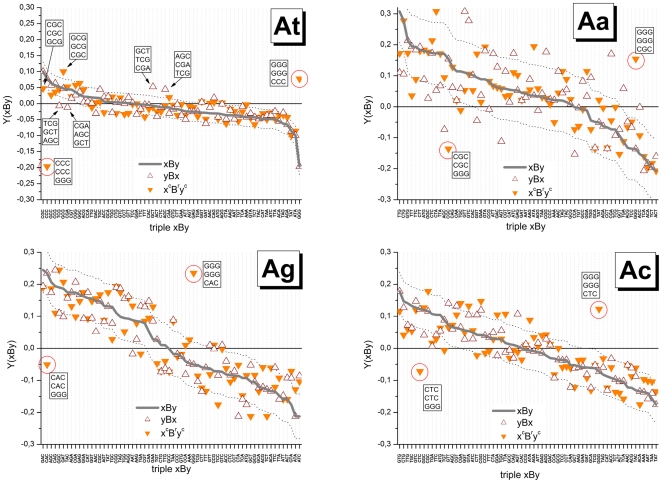
Symmetry relations of triple interactions. The triple sensitivities, Y(xBy), of each interaction groups are ranked in decreasing order and shown by thick lines. For each base-triple three sensitivity values are shown according to Eq. (14) to reveal 3′/5′-asymmetry, Y(yBx), and complementarity, Y(y^c^B^r^x^c^), respectively (symbols are assigned in the figure). The abscissa labels indicate the xBy-triple. The letter-triples in the boxes indicate special triples the sensitivity values of which reveal considerable asymmetry, for example xBy/yBx/y^c^B^r^x^c^ = TCG/GCT/AGC of the At-group. Note that GGG-motifs are highly non-complementary in all four interaction groups. Note also the markedly different widths of the scattering funnels of the different interaction groups given by their standard deviation (see dotted lines and also Table 1) indicating that the stacking terms and/or asymmetry of interactions are differently modulated by the central mismatch (see text). For symmetry reasons part of the asymmetries differences vanish (e.g. 3′/5′-asymmetry of GGG/GGG/CAC).

Figure 5 re-plots the triple sensitivities shown in Figure 4 in decreasing order for each group (see thick line in each panel) together with the values which are re-ordered according to the symmetry-relations Eq. (14) (see symbols). We calculate the scatter width of the symbols about the ranked xBy-triples in terms of their standard deviation which defines a sort of “asymmetry” funnel shown by dashed curves in Figure 5. The widths of the funnels (the respective standard deviations are given in Table 1) characterize the mean asymmetry of the triple interactions of the respective interaction group. Note that for perfect symmetries one expects vanishing funnel widths.

Both, 3′/5′- and complementary asymmetries roughly behave in parallel. They are, by far, smallest for the At-group and largest for the Aa-group which agrees with the ranking of the variability of the triple sensitivities between the groups. Also the SD values roughly agree (see Table 1) which indicates independence of triple sensitivities after symmetry transformation.

Hence, the effect of the central mismatch of the Aa-group is obviously most modulated by stacking interactions and complementary asymmetries among the considered groups causing largest variability of the associated probe intensities. Note that just this type of self-complementary mismatches was selected to design MM probes on microarrays of the GeneChip-type. Our results suggest that this design seems suboptimal because it is associated with a relatively high variability of mismatch stability. The effect introduces additional noise into the MM intensities which intend to correct the PM signals for background contributions.

Examples for symmetry relations are explicitly indicated in Figure 5 (the respective triples xBy/yBx/y^c^B^r^x^c^ are given within the boxes, the abscissa labels indicate the xBy-triple only): For example, the combination AGC/CGA/TCG taken from the At-group shows marked 3′/5′-asymmety beyond the limits of the mean scattering funnel. The data clearly show that the by far largest complementary asymmetries are associated with triple-G motifs in the probe sequence for all interaction groups (see solid triangles surrounded by the circles). They make a contribution of up to 50% to the mean variability of the respective interaction groups (Table 1). Note in this context that the GGG-motifs are characterized by the weakest interactions either among all 64 triples (At-group) or among the 16 triples with a central G (Aa-, Ag- and Ac- groups, see Figure 4). This effect will be further discussed below.

### Adjacent WC pairings

The context of adjacent WC pairs considerably modifies the effect of the central mismatch: For example, the ratio of two triple-sensitivities with a central Cc-mismatch (Aa-group) flanked either by two C's or by two A's is about Y(CCC)/Y(ACA)|_Aa_≈10^+0.2^∼1.6 whereas the respective intensity ratio for the triples with a central Cg-pair (At-group) is only I(CCC)/I(ACA)|_At_≈10^+0.1^∼1.25.

**Figure 6:**
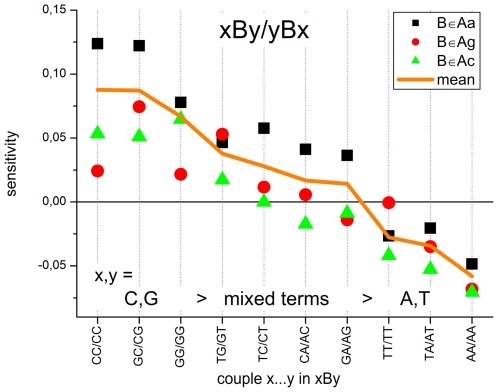
The effect of adjacent WC pairings in triples with a central mismatch. Mean sensitivity values were calculated as averages over triple sensitivities shown in Figure 4 for each Ab-group over the central mismatch. The obtained values characterize the mean effect of the couple xy in the triple xBy. They are ranked with decreasing mean of all three mismatch groups. It shows that x,y = C and G give rise to largest sensitivities and standard deviation about the mean whereas adjacent x,y = A and T cause smaller sensitivities and variability about the mean.

To generalize this result we average the triple sensitivities of each mismatch group over the central base, 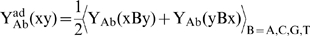. The obtained mean sensitivities characterize the effect of the WC pairings adjacent to the mismatched pairing. The values rank in good agreement with the expected mean stability of single-nucleotide canonical DNA/DNA interactions, C≈G>A≈T [32] (see Figure 6). Note also the small systematic trend between the Ab groups, Aa>Ag≈Ac, and the decreasing variability of the data with decreasing mean.

### Tandem mismatches

**Figure 7:**
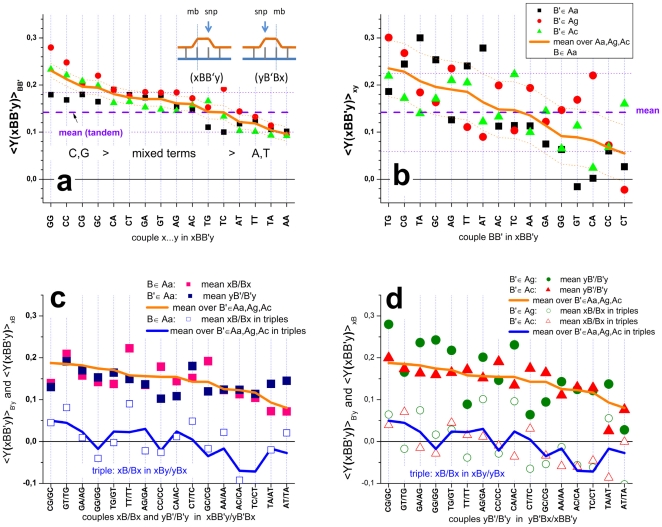
The sensitivities of quadruplets (xBB'y) composed of central tandem mismatches BB' and edging WC pairings, x,y. The quadruplets were analyzed in terms of independent duplets of the WC-couples xy (part a), of tandem mismatches BB' (part b) and of mixed NN-couples xB/Bx and yB'/B'y (part c and d). Note that B refers to the Aa-group whereas B' to the Aa-, Ag- or Ac-group (see legends in the figure). Along the x-axis the respective pairings are ordered with decreasing mean sensitivity which is averaged over the three groups Aa, Ag and Ac of B' (see the thick decaying curve). Part a and b: The central tandem mismatches formed by B and B' cause considerably larger scattering than the adjacent WC pairings formed by x and y. The thin dotted curves running parallel to the thick line illustrate the standard deviation of the dots about their mean (see also Table 1). In part c and d the respective NN-terms derived from the triple motifs with single mismatches (see Eq. (16) and Figure 10 below) are shown for comparison (the open symbols show the NN-terms of the respective interaction groups and the thick blue line their mean value).

Tandem mismatches occur in homozygous-absent duplexes of the MM-probes (MM-G'•G) with SNP offsets δ = +1 and −1 (see Text S1). They consist of a mismatch of the Aa-group at the middle position of the probe sequence and a second mismatch of the Aa-, Ag- or Ac-group at the adjacent SNP position (see the sketch in Figure 7, panel a). The tandem mismatches are analyzed together with the adjacent WC pairs forming the quadruplets (yB'Bx) and (xBB'y) for δ = −1 and +1, respectively (where x, y, B and B' denote the respective nucleotide bases in the probe sequence). According to this convention we ignore the strand direction: B defines the mismatch of the Aa-group and B' the mismatch of the Aa-, Ag or Ac-type and x and y form the edging WC pairings adjacent to B and B', respectively. The need for considering quadruplet-motifs (tandem mismatch and flanking WC pairs) to specify the stability of two adjacent mismatches was discussed previously [34].

We calculate the sensitivities of all possible combinations for each of the three possible options of B' (referring either to the Aa-, Ag- or Ac-group) using the background-corrected intensities relatively to the mean log-intensity of the probes with two mismatches (#mm = 2) with at least one WC pairing in-between, Y_Ab_(xBB'y) = log(I_Ab,#mm = 2,|δ| = 1_(xBB'y))−<log(I)>_#mm = 2,|δ|>1_ (see also part a of Figure 2).

The average values of the obtained sensitivities of the tandem mismatches are positive (see the horizontal dashed lines in part a and b of Figure 7) which reflects their larger stability compared with the double mismatches which are separated by at least one WC pair.

The 16^2^ possible quadruplet combinations were reduced to 2×16 values for each of the three possible pairings of B' by calculating the average either over the edging WC pairings xy or over the mismatches BB', <Y_Ab_(xBB'y)>_xy_ and <Y_Ab_(xBB'y)>_BB'_, respectively. We consider all 16 combinations of xy and BB' in xBB'y because both members of each couple are not equivalent (B'∈Aa, Ag, Ac and B∈Aa). The obtained values thus characterize the effect of the edging base couples xy (part a of Figure 7) and of the mismatch couples BB' (part b) on the corresponding probe sensitivities, respectively. In addition we decompose the quadruplets in two consecutive NN-contributions according to xBB'y→xB+B'y/yB'Bx→yB'+Bx by calculating the averages ½<Y_Aa_(xB)+Y_Aa_(Bx)>_B'y_ and ½<Y_Ab_(B'y)+Y_Ab_(yB')>_xB_ , respectively (see part c and d of Figure 7), which characterize mixed combinations of WC- and mismatched pairings in accordance with the NN-decomposition of the standard triples applied in the next section.

The couples of edging bases x and y cause considerable smaller variability of the probe sensitivities than the couples of adjacent mismatches (compare part a and b of Figure 7). The standard deviations of the latter group exceeds that of the former group roughly by the factor of two (see Table 1). This ratio actually increases to about three if one calculates the scattering about the mean of the three Ab-groups (i.e. the scattering about the decaying line in the figure). Hence, the particular couple of mismatches BB' mainly modulates the intensities of the probes whereas the edging WC pairings give rise to only moderate intensity variations. This result agrees with the properties of triples with a central mismatch discussed above. The main source of probe intensity variation was also attributed to the central mismatch in this case.

Part a of Figure 7 shows that the adjacent WC pairs rank according to x,y = C,G>x = G,C; y = A,T>x = A,T; y = G,C>x,y = A,T and thus in the similar order as the adjacent WC pairs of single mismatches (see previous section and Figure 6). Both sets of mean sensitivities (thick lines in Figure 6 and Figure 7, panel a) correlate with a regression coefficient of R = 0.57.

Part b of Figure 7 indicates that the particular sensitivity value strongly depends on the combination of mismatches. For example, the combination BB' = CT of, on the average, relatively weak stability varies between large and very small sensitivities for C∈Ac and C∈Ag, respectively.

Alternatively, we decomposed the quadruplets with the central tandem mismatch into two consecutive NN-terms as described above (Figure 7, panel c and d). These NN-terms can be compared with NN-terms which were obtained after decomposition of the triple sensitivities into two NN-terms as described in the next section (compare with thick blue lines and open symbols in Figure 7, panel c and d). Both data sets correlate with regression coefficient R = 0.69. This result suggests that quadruplets with central tandem mismatches can be decomposed to a rough approximation into two NN-terms which can be estimated also from triple data.

### Flanking mismatches

Triples with flanking mismatches of the type w(xBy)m (B∈At; “w” and “m” denote a WC- and a mismatched pairing, respectively, i.e. w∈At and m∈Aa,Ag,Ac) were selected according to the scheme shown in Text S1. These triples refer to SNP offset positions |δ| = 2. To assess the effect of the flanking mismatch “m” we compare the log-intensities of the respective probes with the respective values of the neighboring standard triples w(xBy)w without flanking mismatch (offset |δ| = 3),
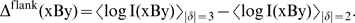
(15)This difference estimates the mean intensity increment of the standard triple without flanking mismatches relative to that with flanking mismatches. Our nomenclature assigns nucleotide ‘y’ to the position adjacent to the mismatch which flanks the triple, (xBy)m. This neighborhood-relation can be realized for the triples (xBy)m and m(yBx), i.e. with the mismatch facing towards the 3′ or the 5′ end of the probe, respectively; and, in addition, in the probe and target sequence according to the complementary condition m(yBx)→(x^c^B^c^y^c^)m (the superscript “c” denotes the WC-complement). These, in total four options (for example (CGT)m, m(TGC), (GCA)m, m(ACG)) are averaged to provide the mean effect of the flanking mismatch adjacent to ‘y’ and ‘y^c^’ on the selected triple.

**Figure 8:**
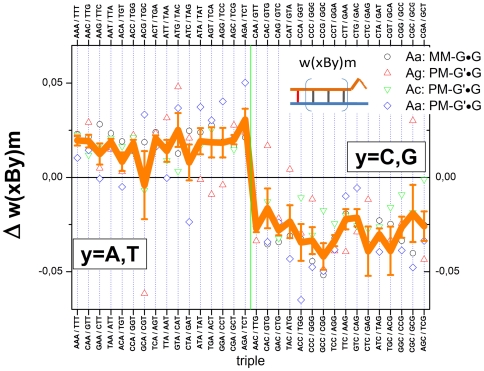
Excess sensitivities of triples with flanking mismatches (Eq. (15)). The respective probes with flanking triples are selected according to Text S1. Neglecting 3′/5′- and probe/target-asymmetries, each value is calculated as mean value over the four triples indicated at the lower and upper x-axes for each mismatch group (symbols; see legend for assignments). The combination of triples shown at the lower axis denote the complements w(xBy)m/w(x^c^B^c^y^c^)m and that at the upper axis m(yBx)w/m(y^c^B^c^x^c^)y. The thick line refers to the total mean over all three mismatch groups m∈Aa,Ag,Ac. The excess values are consistently positive and negative for adjacent y = A,T and y = C,G, respectively.

Figure 8 shows that the obtained mean excess values are consistently negative for y = C,G and positive for y = A,T. Hence, a mismatched pairing either stabilizes or destabilizes the adjacent triple in dependence on the neighboring base y. The effect is however relatively weak and amounts to a few percent of the respective probe intensity.

### Nearest neighbor terms

In analogy with the NN free energy contributions in models describing the stability of DNA/DNA-oligonucleotide duplexes in solution (see [32], [33] and references cited therein) we decompose each triple-averaged sensitivity of each interaction group, Y_Ab_(xBy), into two nearest neighbor (NN) terms, Y_Ab_(xB) and Y_Ab_(By), and two single-base boundary contributions according to
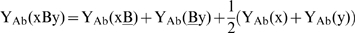
(16)using Single Value Decomposition (SVD) [35]. The underlined letter denotes the central base of the respective triple in the argument of the NN-terms to avoid confusion in symmetry relations discussed below. The single-base boundary terms consider the mean effect of the bases adjacent to the triple. The triple data of each interaction group thus define a system of 64 linear equations which was solved by multiple linear regression to determine in total 8 boundary and 32 NN terms (see also [20]).

We first examined the adequacy of the decomposition (Eq. (16)) in terms of the residual contribution
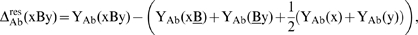
(17)which estimates the degree of additivity of the triple NNN-model, i.e., the reliability of decomposition of the triples into nearest neighbor NN-terms. In the absence of interactions affecting next nearest neighbors, one expects vanishing residuals, Δ_Ab_^res^(xBy) = 0. Especially the propensity of selected sequence motifs for intramolecular folding of the probes and/or the targets and also for the formation of special intermolecular complexes are expected to involve longer runs of subsequent nucleotides causing deviations from the additivity assumption (Eq. (16)).

**Figure 9:**
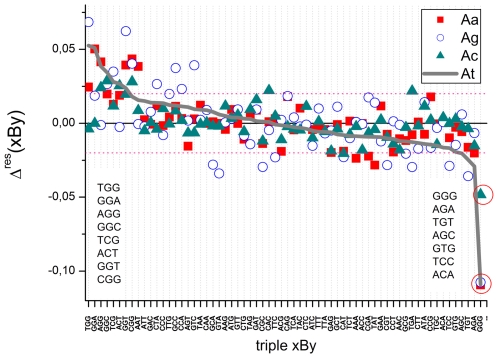
Residual sensitivity after decomposition of the triple sensitivities into NN-terms (Eq. (17)). The symbols refer to the mismatched interaction groups. The triples are ranked with decreasing residual contributions of the At-group. The horizontal dashed lines mark the average standard deviation of the data about the abscissa. The two NNN-lists indicate the largest positive (left list) and negative (right list) residual-values of the At-group. Note that triple GGG provides by far the largest (negative) residual contribution (see red circles). Positive contributions are obtained for triples containing the couple ‘GG’ which indicates that the respective NN-terms underestimate their contribution to the triple sensitivities.

Figure 9 shows the residuals of all 64 triples per interaction group obtained after decomposition of the NNN-terms into nearest neighbor contributions. The standard deviation of each group is considerably smaller compared to that obtained from the asymmetry relations (see Table 1). This result indicates that most of the triples are additive with respect to NN-terms to a good approximation.

However, motifs containing couples of adjacent GG are prone to positive deviations from additivity indicating that the respective GG-term systematically underestimates the contribution of two adjacent guanines to the triple term. On the other hand, runs of three guanines, ‘GGG’, give rise to the strongest negative residual terms of all interaction groups. The triple sensitivities Y_Ab_(GGG) are negative for all interaction groups (see Figure 4). The observed residuals thus again indicate that the respective sum of two GG-terms underestimates their contribution to the absolute value of the triple sensitivity, i.e. 2 |Y(GG)|<|Y(GGG)|. Hence, non-additivity of the considered triples is mainly introduced by GG-couples, the NN-terms of which underestimate their contribution to triple terms containing adjacent GG.

**Figure 10:**
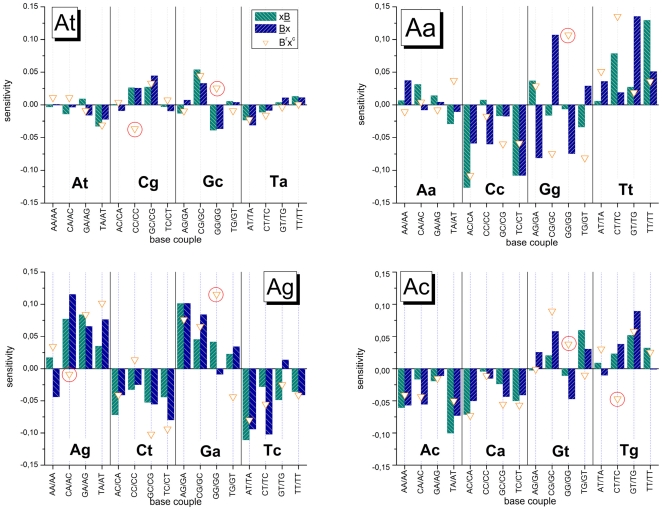
Nearest neighbor (NN) sensitivity terms of the four interaction groups. The NN-terms are calculated via decomposition of the triple terms using SVD (Eq. (16)) where the base couples are ordered with respect to the centre base B of the triples. The base couples are indicated as abscissa labels xB/Bx (left/right bar, respectively). The symbols are the sensitivities after applying the complementary transformation to the NN-terms, xB→B^r^x^c^. NN-terms related to ‘GG’-motifs are indicated by red circles. They strongly deviate from the complementary condition.

Figure 10 separately shows the obtained NN-terms for each interaction group and for each central base pairing of the respective triples. The NN-terms are combined according to the convention xB/Bx (left/right bar) which estimates the 3′/5′ asymmetry with respect to the common base B forming the mismatched pairing in the Aa-, Ag- and Ac-groups. Comparison of the respective left and right bars essentially confirms the 3′/5′-asymmetry data of the triple sensitivities discussed above, namely that the Aa- and At-groups show the largest and smallest asymmetries, respectively. The NN-data in addition reveal that most of the highly asymmetric base couples of the Aa-group (e.g., AC/CA, CC/CC, AG/GA, CG/GC) are associated with guanines and cytosines at the mismatch position.

### Comparison with free energy terms describing duplexing in solution

**Figure 11:**
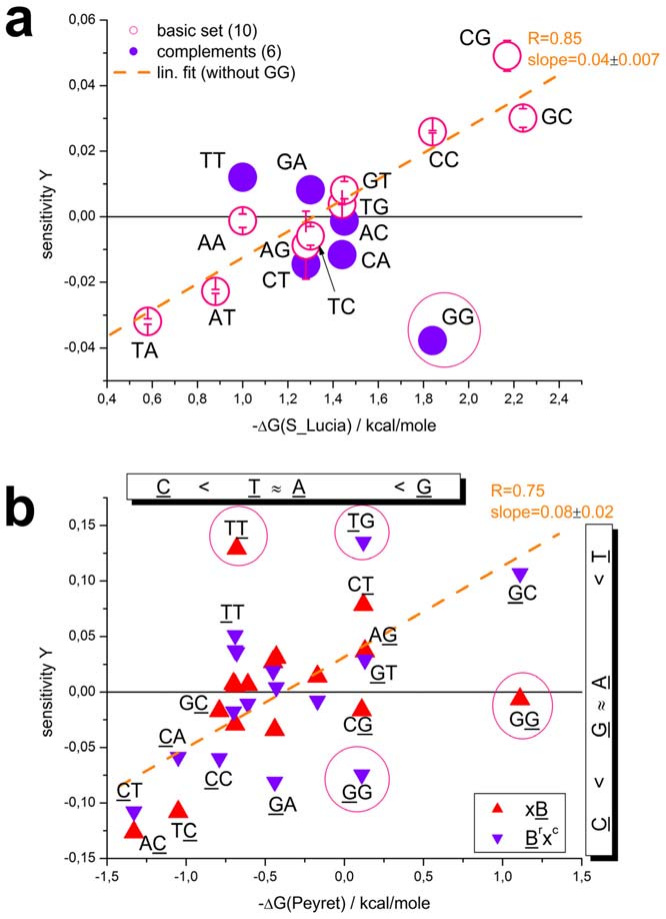
Comparison with solution data. The figure shows the sensitivity NN-terms of the At- (part a) and Aa- (part b) groups obtained in this study (Eq. (16)) with NN-stacking free energy terms for DNA/DNA-duplexes in solution taken from ref. [33] and [31], respectively. The dashed diagonal lines are linear regressions using all NN-data except the double-guanine terms (At-group) and in addition except TT and TG (Aa-group) which are included in red circles (regression coefficients and slopes are given in the figure). Panel a: Each NN-sensitivity of couple XY was calculated as the mean value averaged over the two sensitivities with arguments XY and XY shown in Figure 10. The difference between these paired values is shown by the error bars which typically do not exceed the size of the symbol. The basic set of 10 independent terms is indicated by open circles. Panel b: The complementary couples xB and B ^r^x^c^ are shown by different triangles. Only selected NN-motifs are assigned. The apparent mean stabilities of the mismatched pairings rank differently for chip (see vertical bar) and solution (horizontal bar) data.

The 32 NN-couples of At-groups can be further reduced to 16 NN-terms making use of the symmetry-relation Y_At_(XY)≈Y_At_(XY) which however only applies to the At-group due to the equivalence of the two WC pairings associated with the nucleotide letters. Part a of Figure 11 correlates the obtained 16 averaged terms, Y_At_(XY) = 0.5⋅(Y_At_(XY)+Y_At_(XY)), with the ten NN-free energy terms estimated in solution studies [33]. The data well correlate with a regression coefficient of R = 0.85 if one ignores the GG-couple (see regression line in Figure 11). Its sensitivity value distinctly deviates in negative direction in agreement with the qualitative discussion of the residual contributions given above (see Figure 9). The relatively large difference Y_At_(CC)−Y_At_(GG)>0.06 indicates that the complementarity between CC and GG is clearly disrupted. On the other hand, the sensitivity values of the remaining complementary couples (XY/Y^c^X^c^ = AA/TT, CT/AG, TC/GA, AC/GT and CA/TG; see full and open symbols) are relatively close each to another (mean difference |Y(XY)−Y(Y^c^X^c^)|≈0.01) which justifies utilization of the complementarity condition to a good approximation. The linear regression coefficient slightly improves (R = 0.92) after averaging over the complementary couples. Hence, except GG-motifs, the interactions of canonical WC pairings estimated from the probe intensities of SNP GeneChip microarrays in acceptable agreement correlate on a relative scale with free energies in solution.

Part b of Figure 11 shows an analogous correlation plot for the NN-terms of the Aa-group where the solution free energies were taken from ref. [31]. The 32 NN-sensitivity terms split into 16 basic terms Y_Aa_(xB) (open symbols) and 16 complementary terms Y_Aa_(B ^r^x^c^) (solid symbols). As for the At-group, the double-guanine terms strongly deviate from the regression line and were excluded from the linear fit (R = 0.65). Additional exclusion of double-thymines further increases the regression coefficient (R = 0.75) which indicates satisfactory correlation between solution free energy data and most of the NN-sensitivities. A recent study also reports clear correlation between solution and array estimates of hybridization free energies using a specially designed Agilent microarray containing sets of PM and MM probes with #mm = 1 and 2 mismatches upon duplexing [36].

Note that the mean stability of self-complementary mismatches rank according to CC<TT≈AA<GG in solution but according to Cc<Gg≈Aa<Tt on the chip (see Figure 7). Hence, Gg-pairings apparently loose and Tt-pairings gain stability on the chip. The stability-ranking of the other mismatches except Gt essentially agrees for solution and chip data (see above).

## Discussion

In this study we analyzed the probe intensities taken from a 100k GeneChip SNP array in terms of selected sequence motifs forming well defined WC- and mismatched base pairing in the probe/target duplexes. The particular probe design of these GeneChip SNP arrays enables one to disentangle different sources of intensity modulations such as the number of mismatches per duplex, the particular matched or mismatched base pairings, their nearest and next-nearest neighbors, their position along the probe sequence and the relative position of a second mismatch. As the elementary sequence motif we chose triples of subsequent nucleotides centered about the middle base of the probe and/or about the SNP base and calculate log-averages of the intensities over thousands of probes with identical motifs to average out the effect of the remaining sequence. These averages are measures of the stability of the base pairings formed by the selected triple in the probe sequence with the corresponding base triple in the target sequence. The former triple is defined by the probe sequence whereas the target triple can be deduced from the genotype and the hybridization mode. We analyzed the log-averaged intensities, their difference to selected reference values, the so-called sensitivity, and their variability in subsets of triple-motifs. In addition to triple motifs, we also consider special motifs such as flanking mismatches adjacent to the triples and tandem mismatches which were analyzed in terms of quadruplets including the edging WC pairings.

The first question of our analyses addresses the impact of different interaction motifs on the observed probe intensities. It turns out that

a) the number of mismatches per probe/target-duplexes exerts the largest effect which modulates the intensity. One mismatch is associated with the logarithmic intensity change of −δlogI = 0.5–0.6 which is equivalent with the decrease of the intensity by a reduction factor of about F = 0.3−0.25 per mismatch.

b) the effect of mismatches is strongly modulated by the adjacent WC pairings which give rise to a mean logarithmic increment of ∼δlogI = ±0.1, or equivalently, with an average modulation factor of 0.8<F<1.25 (see Table 1). Selected motifs cause larger log-increments of δlogI = ±0.3 (see Figure 4) which are almost comparable in magnitude with the mean mismatch effect (see a).

c) duplexes with tandem mismatches are more stable than double mismatches which are separated by at least one WC pairing (δlogI≈+0.1 and F≈1.25).

d) flanking mismatches adjacent to the considered triples only weakly modulate their intensities (|δlogI|<0.025; 0.95<F<1.05).

e) the mean variability due to sequence effects in triples of WC pairings is markedly smaller than the effect in triples with a central mismatch (δlogI = ±0.05; 0.9<F<1.1; compare with b).

f) runs of three guanines in the probe sequence forming nominally WC pairings represent a special motif which decreases the intensity to an exceptionally strong extent (δlogI = −0.2−−0.35; F = 0.6−0.45). Also mismatched duplexes with runs of guanines possess relative small intensity values which are virtually incompatible with expected interaction symmetries in DNA/DNA-duplexes.

g) the positional dependence of triple-averaged intensities along the probe sequence is relatively weak (see Figure 3 part a, c and d). The sequence-specific effect progressively disappears towards the ends of the probe sequence at the final 3–5 sequence-positions for most of the motifs. Triple ‘GGG’-motifs partly deviate from this rule: Along the whole sequence they markedly reduce the intensity. In mismatched duplexes one observes the opposite effect at the probe end facing towards the supernatant solution.

h) especially small (e.g., for probes with two mismatches, #mm = 2) and large intensity values are prone to background and saturation effects, respectively (see Text S1). Appropriate background corrections should consider the optical background and partly also non-specific hybridization. Saturation can be considered using the hyperbolic adsorption law (see supporting file Text S1).

Our analyses also address the question whether the number of considered sequence motifs can be reduced by utilizing symmetry relations and/or by decomposing the triple averages into nearest neighbor terms in analogy with interaction models for oligonucleotide duplexes in solution. It turned out that

i) triples of WC pairings (At-group) can be reasonably well decomposed into NN-terms which also meet the complementary condition to a good approximation and correlate well (R = 0.85) with the independent NN-free energy terms derived from duplex-data in solution [32], [33]. GGG-motifs strongly deviate from these properties and must be considered separately.

j) also the triples with a central mismatch (Aa-, Ag- and Ac-group) to a good approximation decompose into NN-terms except special motifs containing at least doublets of guanines. The mismatch motifs partly obey the symmetry relations, however, with larger residual variability compared with WC pairings. Comparison with NN-terms of solution free energies [31] indicates satisfactory correlation for most of the motifs (R = 0.75). Runs of guanines and partly also thymine-containing motifs deviate from the expected behavior in negative and positive direction, respectively.

k) tandem mismatches can be decomposed into two NN-terms referring to a combination of mismatched and WC pairings. These values well correlate (R = 0.59) with the NN-terms obtained from the triple data suggesting to use a unified set of NN-terms (see j). For tandem mismatches one has however to consider their systematically larger stability compared with duplexes containing two mismatches which are separated by at least one WC pairing.

In the following subsections we discuss the physical origin of selected effects more in detail and derive rules for appropriate correction of parasitic intensity errors to obtain unbiased genotyping estimates.

### Relation to thermodynamics

The intensity of microarray probes is directly related to the effective association constant for duplexing, ∼K_duplex_ after correction for parasitic effects (or their neglect, if justified) such as the optical background, non-specific hybridization and saturation (see Eq. (2)). The effective association constant is a function of different reaction constants characterizing relevant molecular processes such as the bimolecular stacking of unfolded probes and targets (P•T, P•P, T•T), and their unimolecular folding propensities (P-fold, T-fold) [13] (see also [37]), i.e.
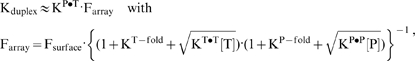
(18)where F_surface_<1 is a factor taking into account surface effects, such as electrostatic and entropic repulsions which effectively reduce target concentrations near the array surface. According to Eq. (18), the effective constant of duplex formation is reduced by the factor F_array_<1 compared with the stacking interaction constant K^P•T^. Folding and/or self-dimerization of probe and/or target become relevant at 
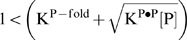
 for the probe (substitute P→T for the target).

Stacking interactions are mainly governed by the pairings formed between the nucleotides in the target and probe and their nearest-neighbors along the sequence. The decomposition of the corrected intensity into different interaction modes associated with single target-types enables assignment of the probe sequence to canonical and mismatched base pairings with the target. We analyzed triple motifs which represent a reasonable choice to study stacking interactions on an elementary level. Note that also the reduction factor F_array_ depends on the probe and target sequences, however in a more subtle fashion because, for example, folding reactions comprise longer sequence motifs.

The duplex-association constants can be multiplicatively decomposed into a triple-related factor which modulates the total (average) contributions
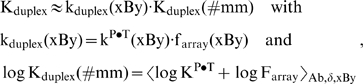
(19)where we use the notations introduced above. The triple related terms are denoted by lower case letters. The overall mean of the association constant mainly depends on the number of mismatches in the duplex, #mm. The modulation factor and the mean value are decomposed into stacking and array terms using Eq. (18). Hence, the effective duplex association constant decomposes into a series of nested factors which consider triple motifs, stacking interactions and array specifics in different combinations.

Comparison with Eq. (8) and considering the direct relation between the corrected intensity and K_duplex_ provides the relation between the analyzed observables and the binding constants,
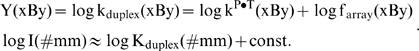
(20)The logarithm of the association constant defines the stacking free energy of the duplex, ΔG^P•T^∼−logK^P•T^, which applies also to the triple terms, i.e., ΔΔG^P•T^(xBy) = ΔG^P•T^(xBy)−<ΔG^P•T^>∼−logk^P•T^. With this definition and Eq. (20) one finds
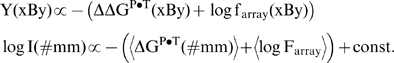
(21)Hence, the triple-averaged sensitivities are related to the deviation of the stacking free energy due to the considered triple from its mean value. This increment is however distorted by an “array”-term caused by folding, self-duplexing of target and probe and by specific surface effects. The former contributions are also functions of the sequence position of the chosen triple which is not explicitly expressed in Eq. (21) for sake of convenience. Note also that imperfect probe synthesis potentially reduces the real length of the oligomers in a motif-specific fashion with possible consequences for the observed triple sensitivities [13].

The sensitivity and free energy change into opposite directions, i.e. larger stability of interactions is associated with larger Y but smaller (more negative) ΔG. After decomposition into NN-terms we found acceptable correlation between the estimates from chip data and solution data taken from the literature for most of the motifs (see Figure 11). We conclude that chip effects are of inferior importance on the average (i.e. 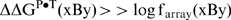). Stacking free energies therefore well reproduce the relation between the particular terms on a relative scale.

The proportionality constant in Eq. (21) is estimated by the slope of the regression lines in Figure 11. Their values are with (0.4–0.8)⋅10^−1^ roughly one order of magnitude smaller than the proportionality constant predicted by the thermal energy ∼1/(RT⋅ln10)≈0.7 (T≈40°C). We previously argued that non-linear (in logarithmic scale, as, e.g., predicted by Eq. (18)) and sequence dependent contributions to log(f_array_(xBy)) can cause proportionality constants less than unity [13]. Sequence-independent sources of intensity variability such as the length-dependent yield of the genomic targets after PCR-amplification [9], [38] not-considered here are potential causes of the downscaling of the proportionality constant. Interestingly, the proportionality constant obtained for the mismatched pairings (Aa-group) exceeds that for the WC pairings (At-group) by the factor of two (compare part a and b of Figure 11). This difference suggests that the larger sensitivity-response of the probes to mismatched pairings (compared with WC pairings) is not simply related to the variability of the respective stacking free energies but includes other effects related to the array technology.

### Mismatches

**Figure 12:**
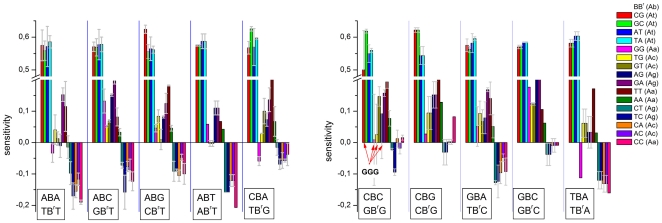
Stability of mismatch motifs. Relative stabilities of the 10 possible contexts of complementary triples containing the 16 possible central base pairings (mismatches or Watson-Crick base pairs, see legend in the figure). The sensitivities of the pairs of complementary triples xBy/y^c^B^r^x^c^ (B^r^ = B') are averaged using the triple data shown in Figure 4. The error bars indicate the difference between the individual values and thus they quantify the deviation from complementary symmetry. The form of the bar diagram was chosen in correspondence with Figure 3 in ref. [31] which ranks the stacking free energies of each triple in solution-duplexes with decreasing stability (from left to right for each triple). The mean log-intensity increment of one mismatched pairing (see Figure 2) was added to the triple-values of the At-group to compare the stabilities of WC- and mismatched pairings in a unique scale. The sensitivities of the four triple-combinations in the GGG-context are exceptionally small (see the red arrows).

The stabilities of most of the mismatched pairings (Eq. (12)) rank in similar order as the results of previous chip and solution studies (Eq. (13)). Figure 12 shows the detailed stability trend in all 10 possible contexts of complementary triples with all 16 possible pairings of BB' (accordingly, the couples BB' refer to the pairings B•b^r^ and B'•b'^r^ with b^r^ = B' and b'^r^ = B, respectively). Our figure was designed similar to Figure 3 in ref. [31] which ranks the central bases according to its mismatch stability in solution (Eq. (13)). Essentially two groups of larger and weaker stabilities can be clearly distinguished for BB': (TT,GA,GA;GT,TG,AA,GG)>(CT,TC,CA,AC,CC), respectively (see also the detailed ranking in Eq. (12)). Hence, mismatched pairings formed by cytosines are consistently of weaker stability. Most of the triples are modulated by the nearest neighbors of the central base (x…y) which follows the mean trend shown in Figure 6 (i.e., (x…y) = G,C>A,T). As an exception, adjacent WC pairings however only weakly affect the triples with the central mismatches BB' = TT and GA.

The stability of mismatched pairings is governed by the propensity of the paired nucleotides to form hydrogen bonds (e.g., two bonds (T, A) versus three bonds (G, C) in canonical WC pairings), by steric factors such as the size of the aromatic moiety (one ring of the pyrimidines (C,T) versus two rings of the purines (G, A)) as well as stacking effects associated with nearest neighbors.

Stable mismatched base pairs such as GT or GA form two H-bonds and only slightly disrupt the structure of the oligonucleotide-DNA duplex. In particular, the former purine/pyrimidine mismatch GT is usually slightly more stable than the latter purine/purine mismatch GA because a two-ringed guanine better fits with a single-ringed thymine than with a double ringed adenine [30]. On the other hand, unstable mismatched base pairs such as CT or CA significantly disrupt the duplex structure due to the small size of the pyrimidine/pyrimidine pairing or the disability to form at minimum two H-bonds because of the lack of imino protons [30]. Also the self complementary single ringed CC mismatch has a low stacking propensity and forms only one H-bond. This rationalizes the low stability of the mismatches formed by cytosines in agreement with our chip data.

The second self complementary single ringed TT mismatch with low stacking propensity is, in contrast to CC, however stabilized by two H-bonds. The two purine/purine self complementary mismatches GG and AA have a relatively high stacking potential and form either two (GG) or only one (AA) H-bond. One expects therefore the stability-series AA≈TT<GG which is confirmed in solution experiments [31] but disagrees with our chip data and that of others [15] (see also Eqs. (12) and (13)). Especially GG mismatches are apparently much less stable than expected. An analogous low stability of GG mismatches on microarrays compared with solution data was reported for DNA/RNA hybridizations [25]. It has been concluded that thermodynamic properties of oligonucleotide hybridization are by far not yet understood and not suited to assess probe quality.

### Poly-guanine motifs

Consideration of the neighboring bases shows that the apparent low stability of Gg-mismatches is accompanied with triple G-motifs in the probe sequence. These runs of guanines are associated with low intensities in triples with both, central WC- (At-group) and mismatched (Aa-, Ag- and Ac-group) pairings. The stability of central Gg-pairings in the context of adjacent ‘non-G’-bases, on the other hand, roughly agrees with the predictions from solution data (see Figure 11).

Our analyses reveal the following effects of triple-G on the observed probe intensities:

- The GGG-effect is non-complementary, i.e. the complementary triples (e.g. CCC for perfect matches) don't show exceptionally small intensities as probes with GGG do.
- Exceptional small intensities are also observed for triple-G with central mismatches independent of the nominal pairing of the central base (see the arrows in Figure 12 which indicate the GGG-associated motifs BB' = CG, GG, TG, AG in CBC/GB'G).
- The effect is non-additive, i.e. the intensity drop due to GGG is inconsistent with the decomposition into GG-contributions in the context of all triple-motifs.
- The effect depends on the sequence position being typically smaller near the ends of the probe sequence (see Figure 3).
- For probes with one mismatched pairing one observes, in contrast to (iv), that terminal GGG at the solution end of the probes gain intensity, i.e. the sign of the effect reverses compared with the remaining sequence positions.
- The intensity drop due to one triple-G corresponds roughly to 50% of the intensity loss due to one mismatched pairing (see Figure 3).

The observations (i) and (ii) strongly indicate that the triple-G effect is not associated with the nominal base pairings deduced from the binding mode because otherwise one expects equal intensity changes for complementary sequence motifs. Observation (iii) indicates that the effect exceeds the range of stacking interactions with the nearest neighbors. Observation (vi) shows that the magnitude of the effect is relatively large compared with the variability due to other base-specific effects but smaller than the variability due to single mismatches.

**Figure 13:**
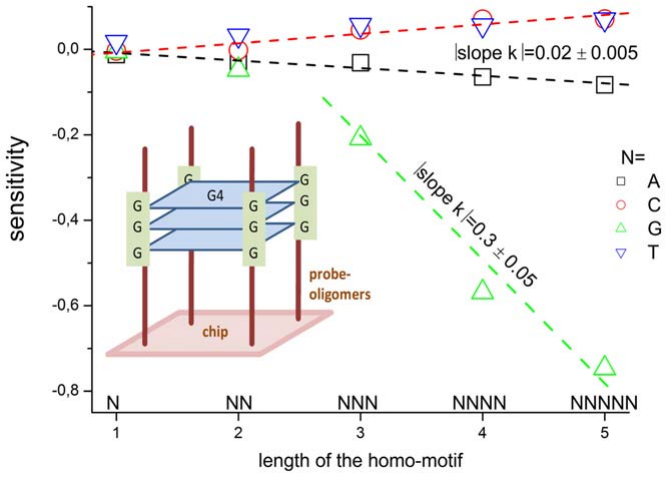
Sensitivities of runs of identical bases. The sensitivity values are averaged over all sequence positions of homo-motifs of length 1 to 5 of homozygous present probes (PM-G'•G, see also Figure 3). Adenines, cytosines and thymines follow straight lines the slope of which is related to the mean stability increment per additional WC pairing in the runs. For guanines the absolute value of the slope drastically increases by more than one order of magnitude for longer poly-G runs exceeding two adjacent G. This effect is attributed to the formation of stacks of at minimum three G-tetrads (G4, see the sketch within the figure which illustrates the structure of a parallel quadruplex formed by four neighbored probe oligomers with GGG-runs at the same sequence position; they are assumed to aggregate into three G4-layers).

To get further insight into the properties of poly-G motifs we calculated the mean sensitivity for runs of identical bases of length one to five, e.g. G, GG,…,GGGGG averaged over all sequence positions of homozygous-present PM-probes (PM-G•G, see Figure 13 and also Figure 3). The sensitivities of all considered runs fit along straight lines with similar absolute values of their slope for adenines, thymines and cytosines (see Figure 13). The slope characterizes the mean sensitivity increment per nucleotide in the run which, in turn, estimates the stability gain (or loss) upon formation of one additional WC pairing in the probe/target duplexes compared with the mean stability of all canonical base pairings. The absolute value of the increment agrees roughly with that of the other bases for single- and double-G (see Figure 13). It however steeply increases for poly-G of length greater than two by more than one order of magnitude. Obviously this change of the slope cannot be attributed to the incremental effect of additional WC pairings in agreement with observations (i) and (ii) but, instead, it presumably reflects the formation of another structural motif accompanied with an increased intensity penalty per additional guanine per run.

Previous studies also reported abnormal intensity responses of probes containing multiple guanines in a row (called G-runs or G-stacks) compared with other probes in different chip assays including Affymetrix expression and SNP arrays [9], [17], [39]–[41]. It was found in agreement with our results that the effect is asymmetric with respect to complementary C-stacks [40], [41] and depends on the sequence position of the stack with a very strong amplitude at the solution-end position [41]. Note that on expression arrays poly-G containing probes show the opposite tendency as on the studied SNP arrays: They shine relatively bright with intensities exceeding the expected signal level [41], [42]. This opposite trend of abnormal strong intensities is associated with non-specific hybridization [41].

The structural rationale behind the poly-G effect has been concordantly assigned to the propensity of poly-G motifs to arrange into stacks of stable molecular bundles of guanine tetrads. These structures potentially affect the efficiency of oligonucleotide synthesis and/or the hybridization of the probes to their target sequences accounting for the abnormal performance of G-runs on the array [17], [39]–[41]. Each G-tetrad is held together by eight Hoogsteen-hydrogen bonds and further stabilized by monovalent cations reducing the electrostatic repulsion between the nucleotides. At minimum three of such planar G-tetrads usually stack together forming very stable complexes via re-folding of one DNA-strand with several poly-G motifs [43], [44] or via aggregation of several DNA-strands with one poly-G motif in each of them (parallel G-quadruplexes, see the sketch in Figure 13). It has been conclusively argued that probe oligomers in close proximity containing poly-G motifs at the same sequence position are prone to aggregate into such parallel G-quadruplexes in the crowded conditions on the surface of high density microarrays [17], [45]. The length of 25-meric probes (∼22 nm) largely exceeds the average separation between neighboring oligonucleotides on such arrays (∼3 nm) which enables complexation of four adjacent probe strands as schematically illustrated in Figure 13. The onset of the stronger sensitivity decrement per additional guanine for triple-G motifs shown in Figure 13 supports the hypothesis that tree layers of G-tetrads represent the minimum motif for stable G-quadruplexes.

As mentioned above, there are two dimensions which potentially affect the performance of probes containing poly-G motifs: firstly, their ability to be correctly synthesized on an array, and secondly the ability of correctly synthesized probes to bind its target.

Let us discuss the first option. The GeneChip arrays are fabricated by *in situ* light-directed combinatorial synthesis on the surface of the array which is prone to produce 5′-truncated products but not internal deletions [46]–[48]. On can suggest that the synthesis yield per nucleotide is reduced in poly-G runs of length greater than two compared with the average synthesis yield possibly because the formation of G-quadruplexes between neighboring probes affects photo-deprotection of the partly synthesized oligonucleotides. As a result of incomplete synthesis the oligonucleotide features are contaminated with probe sequences which are truncated at the nominal position of the poly-G motif. The probability and thus also the number of such truncated probes is expected to increase with the length of the poly-G motif according to the synthesis yield per additional guanine. Truncated probes of length less than 22−20 nucleotides can be assumed to act as weak binders for the targets. Their binding affinity roughly refers to that of full-length probes with more than two mismatches (see Figure 2b and also ref. [13]). The truncated oligomers only weakly contribute to the intensity of the probe spots in mixtures with full length probes at low and intermediate target concentrations. As a result, the observed intensity drop of poly-G containing probe sequences is the result of the reduced number of full length probe oligomers in the respective probe spots. Their fraction can be approximately estimated by assuming proportionality between the intensity drop and the remaining number of full length probes ∼10^Y(GGG)^≈0.4–0.5 for GGG motifs (with Y(GGG) = −0.2…−0.3; see Figure 2b and Figure 13). This fraction is equivalent with the effective synthesis yield per additional G of 40%–50% which roughly halves the number of remaining full length probes according to our data. The general effect of incomplete probe synthesis on the hybridization of microarrays has been discussed in refs. [49] and [13].

Also the second option of modified target binding to correctly synthesized probes provides a tentative explanation of the GGG-effect [45]. It assumes that complex formation between the probe oligomers effectively blocks the involved probe strands and this way reduces the amount of free binding sites accessible for the targets with consequences for their effective association constant which is expected to decrease (see Eq. (18)). The probe-probe interaction term in Eq. (18) assumes simply bimolecular interactions between the probes. Substitution by an appropriate higher-order interaction term which considers the stoichiometry of quadruplex formation, the proximity relations and the fixation of the probes on the chip-surface is expected to modify the respective contribution but leaves the expected trend unchanged.

Note that both discussed potential interpretations of the GGG-effect give rise to a common cause of the observed small intensity values, namely the reduced number of available binding sites for target binding either via truncation or via complexation of part of the probe oligomers. Both interpretations are compatible with our observations (i) and (ii) because the reduced amount of full-length probes and also probe-probe complexes are independent of the respective complementary target sequence upon allele-specific hybridization and independent of the respective mismatched target motif upon cross-allelic hybridization. Also the onset of the increased sensitivity increment per additional guanine for triple-G motifs shown in Figure 13 supports both hypotheses because stable G-quadruplexes of the probes are assumed to affect synthesis and hybridization as well.

Tethering of the involved oligonucleotides to the surface and zippering effects towards both ends of the probes are expected to modify their propensity for G-tetrad formation in a positional dependent fashion in analogy with the positional dependence of base pairings in probe/target dimers [9], [13], [19], [50]–[52]. This trend provides a rationale for effect (iv). Note however that probe-probe interactions modulate target binding via the array-factor F_array_<1 (Eq. (18)). The GGG-profile of homozygous-absent probes (PM-G'•G, see part d of Figure 3) shows the typical characteristics of the mismatched pairing in the middle of the sequence. This result indicates that a certain fraction of the oligomers of the respective probe spot form specific dimers with the cross-allelic or allele-specific target as expected for the respective hybridization mode. This result is in agreement with both hypotheses discussed because incomplete synthesis and probe-probe complexes reduce but not prevent specific hybridization.

The suggested mechanisms explain the decreased intensity of probes containing runs of consecutive guanines. The effect (v) however seems puzzling because terminal poly-G's increase the intensity of the respective probes, instead. On expression arrays one even observes much stronger intensity gains for poly-G containing probes [41], [42]. This opposite trend of abnormal strong intensities is clearly associated with non-specific hybridization. We suggest that G-rich probes are able to form G-quadruplexes of different stoichiometry with non-specific targets containing longer runs of guanines in a positional dependent fashion with a strong bias towards the solution end of the probe. For SNP arrays the relative contribution of non-specific hybridization is relatively weak compared with expression arrays (see Text S1), which explains the relatively weak effect of bright poly-G motifs near the solution end of the probe sequences. Also the fact that effect (v) becomes evident only for relatively weak signals of probes forming at minimum one mismatched pairing is compatible with an additive contribution due to non-specific binding (Eq. (2)). At larger probe intensities, non-specific binding becomes less important compared to specific binding. For completeness we notice that Upton et al. suggested an alternative mechanism which increases the intensity of poly-G containing probes via local opening of regions in the vicinity of quadruplexes [17].

In summary, our data support the hypothesis that runs of consecutive guanines facilitate the formation of stable G-quadruplexes between neighboring probes which in final consequence reduce the number of probe oligomers available for target binding via two alternative mechanisms, firstly, the reduced synthesis yield of full length probes and/or, secondly, the formation of complexes of neighboring full-length probes. Both hypotheses are compatible with the observed intensity drop of probes containing runs of guanines on SNP arrays.

GGG-runs are relatively common on SNP arrays: About 11% of all probes on the studied 100k GeneChip SNP arrays contain at minimum one triple GGG motif and nearly 30% of the allele-sets contain at minimum one of these probes. We conclude that the discussed effect cannot be neglected in appropriate correction methods.

### Correcting probe intensities for sequence effects

The SNP-specific sequence bias transforms into systematic errors of the genotyping characteristics derived from the signals of single probes. Note that the sequence-context of a partial SNP and consequently also the respective bias is essentially very similar for all probes of a selected probe set addressing the same SNP. As a consequence, the averaging of the probe signals into set-related allele values only weakly reduces the systematic signal error after the summarization step. SNP arrays differ in this respect from expression arrays where the sequences of the set of probes interrogating the expression of the same gene or exon can be chosen independently to a larger degree.

One central task of the preprocessing of signals of SNP probes is consequently their correction for sequence effects and in particular for SNP-specific biases. The detailed presentation and verification of an appropriate algorithm is beyond the scope of the present work and will be given elsewhere. The results of our systematic study however enable to identify relevant sequence motifs which significantly modulate the probe intensities. The intensity contributions of such motifs constitute the building blocks of an appropriate intensity model. In particular our results suggest the following rules for sequence correction of SNP probe intensities:

- Sequence effects due to WC pairings between probe and target are well approximated using nearest-neighbor (NN) motifs in analogy with accepted NN-free energy models for oligonucleotide-duplexing in solution [33].
- The anisotropy of probe/target interactions due to the fixation of the probes at the chip surface and end-opening (zippering effects) [13], [49] requires the consideration of the positional dependence of the interactions in a motif-specific fashion, i.e. separately for each NN-combination of nucleotide letters. The assumption of a generic shape function which applies to all motifs seems suboptimal [9], [53].
- The modulation of probe intensities by mismatched pairings can be considered using triple-motifs which consist of the central mismatch and the two adjacent WC pairings.
- Nominal base pairings according to (i) and (iii) can be deduced from the hybridization mode of the respective probes which, in turn, provides selection criteria of the probes for parameter estimation. The mean intensity penalty owing to one and two mismatches can be estimated from the respective class of probes.
- Runs of triple guanines (GGG) represent a special motif which markedly modulates the intensities of the respective probes. The underlying effect does not originate from probe/target (pairwise) interactions but obviously results from the formation of collective complexes presumably of four neighboring probes. Therefore it affects essentially all probes with triple G-motifs independently of the hybridization mode.
- Also tandem mismatches represent a special motif of MM-probes with a modified intensity penalty compared with other MM-probes possessing two mismatches with at least one WC pairing in-between. This sequence effect can be taken into account in a first order approximation by decomposing the quadruple formed by the tandem mismatch and the two adjacent WC pairings into two NN-terms referring to a WC- and a mismatched pairing each, or more roughly, by explicitly considering the two adjacent WC pairings.
- The shift of mismatch motifs by a few sequence positions about the middle base of the probe and the effect of flanking mismatches adjacent to triples with a central mismatch can be neglected to a good approximation.
- Background intensity contributions (optical background and “chemical” background due to non-specific hybridization) should be considered especially for probes forming at least one mismatched pairing.

Established preprocessing algorithms for GeneChip SNP arrays explicitly consider the mean intensity penalty per mismatch [54], [55] or, in addition, the single-base-related positional effect [56]. The authors of the latter work conclude from their results that, after correction, ‘…the sequence effect is reduced but can be further improved’. Our results clearly show that effects which are not taken into account in this model, namely the particular mismatch and its sequence context, the contribution of nearest neighbor stacking interactions and of triple-G runs, considerably modulate the probe intensities. We expect that their explicit consideration will further improve genotyping based on SNP microarrays.

Our present analysis has focused on sequence effects. Note for sake of completeness that an elaborated correction algorithm should also consider additional sources of intensity variation not taken into account here, such as the fragment length and the GC-content of the targets [38], [56] and non-linear effects due to saturation of the probes at large transcript concentrations [23], [57], [58], non-specific hybridization [10] and/or bulk depletion of the targets [59], [60].

### Summary and Conclusions

Single mismatched pairings formed in cross-allelic probe target duplexes and runs of poly G-motifs in the probe sequence are, with the exception of the number of mismatches per duplex, the main sources of signal variability on SNP arrays. These effects must be considered in appropriate calibration methods of the probe intensities to improve the accuracy of genotyping and copy number estimates. The poly-G effect seems to be related to the crowded arrangement of probes on high density oligonucleotide arrays which facilitates the formation of G-quadruplexes between neighboring probes and this way reduces the amount of free probes available for target binding either via incomplete synthesis of full length oligomers and/or via complexation of full length probes. The probe/target interactions on the chip can be decomposed into nearest neighbor contributions which in most cases well correlate with the respective free energy terms describing DNA/DNA-interactions in solution. The effect of mismatches is about twice as large as that of canonical pairings for unknown reasons. Triple-averaging represents a model-free approach to estimate the mean intensity contributions of different sequence motifs which can be applied in improved calibration algorithms to correct signal values for sequence effects.

## Supporting Information

Supporting Text S1 Hybridization modes and base pairings for probe selection. The supporting text provides an overview about the hybridization modes, probe attributes and interaction groups;about base pairings in probe/target duplexes at the middle and SNP position of the probe sequences; and how probes are selected for triple-averaging (including the ‘hook’ criteria and background correction).
(0.44 MB PDF)
