# Supplementary material for: Mismatch and G-Stack Modulated Probe Signals on SNP Microarrays
Source: PLoS One. 2009 Nov 17;4(11):e7862. doi: 10.1371/journal.pone.0007862 (PMC2775684; doi:10.1371/journal.pone.0007862)
Supplement: Supporting Text S1 — Hybridization modes and base pairings for probe selection. The supporting text provides an overview about the hybridization modes, probe attributes and interaction groups;about base pairings in probe/target duplexes at the middle and SNP position of the probe sequences; and how probes are selected for triple-averaging (including the ‘hook’ criteria and background correction). (0.44 MB PDF) [file pone.0007862.s001.pdf]

Supporting Text S1

Mismatch- and G-stack modulated probe signals on SNP microarrays

Hans Binder[1*], Mario Fasold[1], Torsten Glomb[1]

[1] Interdisciplinary Centre for Bioinformatics, Universität Leipzig, Leipzig, Germany

Numbering of reference refers to the reference list of the main paper

Hybridization modes and base pairings for probe selection

1. Hybridization modes, probe attributes and interaction groups

Hybridization mode	Probe attributes			Interaction groups				
	type	SNP offset δ[1]	base position[1]	Ab-group [2] At	Aa	Ag	Ac	no. of mismatches #mm[3]
Specific (S) P-G•G	PM	all	mb	x				0
		all	SNP	x				
	MM	≠0	mb		x			1
		=0	mb/SNP			x	x	
		≠0	SNP	x				
cross-allelic (C) P-G'•G	PM	≠0	mb	x				
		all	SNP		x	x	x	
	MM	=0	mb/SNP			x	x	
	MM	≠0	mb		x			2
		≠0	SNP		x	x	x	

[1] Base pairings formed at the center position of the 25meric probe sequence (mb…middle base) or at the SNP position (SNP) which is offset by δ base positions relatively to the center position. The mb- and SNP positions are consequently identical for δ=0.

[2] Base pairings are classified into four Ab-groups (b = a,t,g,c) as follows: At-group (At, Ta, Gc, Cg); Aa-group (Aa, Tt, Gg, Cc); Ag-group (Ag, Tc, Ga, Ct); Ac-group (Ac, Tg, Gt, Ca). Lower case letters refer to the target.

[3] Number of mismatches per probe/target duplex

2. Base pairings in probe/target duplexes at the middle and SNP position of the probe sequences [a]

Position	SNP offset	SNP type	PM-base B	Base pairing Bb S-mode (P-G•G) PM	Base pairing Bb S-mode (P-G•G) MM	Base pairing Bb C-mode (P-G'•G) PM	Base pairing Bb C-mode (P-G'•G) MM	Probes [b] number	Probes [b] percent
k=13 (mb)	$\delta \neq 0$		T	*At* Ta	*Aa:* Aa	*At:* Ta	*Aa:* Aa	52,940	26.2
			A	*At:* At	*Aa:* Tt	*At:* At	*Aa:* Tt	52,800	26.2
			C	*At:* Cg	*Aa:* Gg	*At:* Cg	*Aa:* Gg	33,008	16.3
			G	*At:* Gc	*Aa:* Cc	*At:* Gc	*Aa:* Cc	33,627	16.7
							total	172,375	85.4
k=13+δ (SNP)	$\delta \neq 0$	[A/C]	T/G	*At:* Ta/Gc		*Ag:* Tc/Ga		14,683	7.3
		[G/T]	C/A	*At:* Cg/At		*Ag:* Ct/Ag		11,224	5.6
		[A/G]	T/C	*At:* Ta/Cg		*Ac:* Tg/Ca		60,547	30.0
		[C/T]	G/A	*At:* Gc/At		*Ac:* Gt/Ac		60,585	30.0
		[A/T]	T/A	*At:* Ta/At		*Aa:* Tt/Aa		9,273	4.6
		[C/G]	G/C	*At:* Gc/Cg		*Aa:* Gg/Cc		16,063	8.0
							total	172,375	85.4
k=13+δ (mb/SNP)	$\delta = 0$	[A/C]	T/G	*At:* Ta/Gc	*Aa:* Aa/Cc	*Ag:* Tc/Ga	*Ac:* Ac/Ca	2,537	1.3
		[G/T]	C/A	*At:* Cg/At	*Aa:* Gg/Tt	*Ag:* Ct/Ag	*Ac:* Gt/Tg	1,956	1.0
		[A/G]	T/C	*At:* Ta/Cg	*Aa:* Aa/Gg	*Ac:* Tg/Ca	*Ag:* Ag/Ga	10,393	5.1
		[C/T]	G/A	*At:* Gc/At	*Aa:* Cc/Tt	*Ac:* Gt/Ac	*Ag:* Ct/Tc	10,265	5.1
		[A/T]	T/A	*At:* Ta/At	*Ac:* Ca/Gt	*Aa:* Tt/Aa	*Ag:* Ct/Ga	1,597	0.8
		[C/G]	G/C	*At:* Gc/Cg	*Ac:* Ac/Tg	*Aa:* Gg/Cc	*Ag:* Ag/Tc	2,777	1.4
							total	29,525	14.6

[a] interaction groups (At, Aa, Ag, Ac) are indicated in leading cursive letters. Note that the probes interrogate each SNP on its sense and antisense strand with mutually complementary sequences. Consequently pairs of complementary letters B and B^c are realized in each probe set giving rise to different combinations of base pairings in the PM and MM probes.

[b] only probes referring to homozygous SNP loci are selected (41,629 out of 58,960 total loci, ~70.1%) and used in further analysis. Note that the probes with $\delta \neq 0$ (85.4% of all used probes) are used twice, considering the sequence motifs about the middle base (k=13) and about the SNP base (k=13+δ). The remaining 14.6% of probes refer to $\delta = 0$. The probes with offset $\delta \neq 0$ split into 27.6% (55,634) with $|\delta|=1$; 14.2% (28,742) with $|\delta|=2$; 14.0% (28,355) with $|\delta|=3$ and 29.5% (59,644) with $|\delta|=4$.

3. Probe selection for triple-averaging

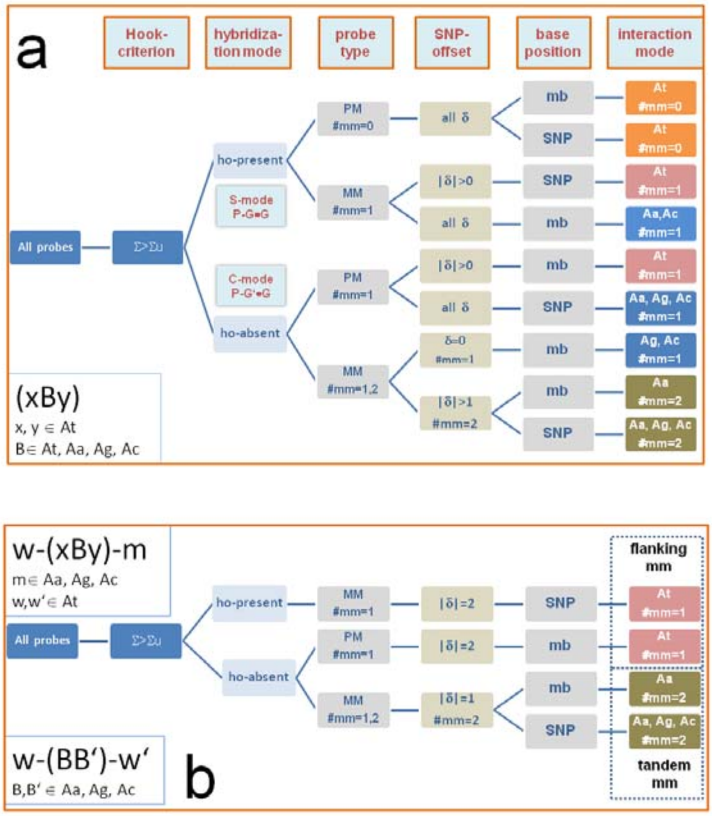

Standard triples (xBy) are selected according to the scheme shown in part a: The interaction mode of the center base of the triple is defined by the chosen hybridization mode, the probe attributes (type, offset) and the position of 'B' (SNP- or the middle base, mb) in the probe sequence. The interaction mode determines the base pairing formed by 'B' with the target according to one of the four Ab-groups, At, Aa, Ag, Ac (see the Tables above), and the total number of mismatches per probe/target duplex, #mm. Part b shows special selections of triples with one flanking mismatch or of tandem mismatches.

4. *'Hook' criteria for probe selection*

Selection criteria considering non-specific hybridization are chosen from the hook-plot of the chip-data (see ref. [5,6] and also the figure). Briefly, the intensities of each probe pair are transformed according to $\Delta = \langle \log(I^{PM}/I^{MM}) \rangle_{allele\text{-}set}$ and $\Sigma = 0.5 \langle \log(I^{PM} \cdot I^{MM}) \rangle_{allele\text{-}set}$. (the angular brackets denote averaging over the respective allele-set), plotted into Δ-versus-Σ coordinates and smoothed using a sliding window of ~ 500 data points. Probe-sets with relatively large contribution of non-specific hybridization, $x^{P,N} > 0.5$ (see Eq. (5)), are characterized by small coordinate-values Σ and Δ. Both coordinates increase with decreasing x^N and level-off at a peak for vanishing contributions of non-specific binding, $x^{P,N} \approx 0$ (see the figure below).

The logarithmic-fraction of the probe-intensity due to non-specific hybridization can be estimated using the coordinate differences with respect to the starting point of the hook curve [6]

$$\log x^{P,N} \approx -\left((\Sigma - \Sigma_{start}) \pm \tfrac{1}{2}(\Delta - \Delta_{start})\right) \quad , \qquad \text{(E1)}$$

where the sum and the difference refer to P=PM(+) and MM(-), respectively. The fraction $x^{P,N}$ depends on the probe type with $x^{PM,N} < x^{MM,N}$ for Σ=const. Practically, a threshold of $(\Sigma - \Sigma_{start}) > 0.7$ is applied to obtain allele sets with an average nonspecific intensity contribution of less than 20%, i.e. $\langle x^N \rangle_{allele\ set} < 0.2$ with $\langle \log(x^N) \rangle_{allele\text{-}set} = 0.5 \langle \log(x^{PM,N}) + \log(x^{MM,N}) \rangle_{allele\text{-}set}$. This implies that the selected allele sets originate at least to 80% either from specific or cross-allelic hybridization.

Note that the hook-plots obtained from SNP arrays lack the horizontal starting range observed typically for expression arrays as a characteristic signature of "absent" probes without complementary targets. Non-specific hybridization to a smaller degree contributes to the signal intensities of SNP arrays compared with expression arrays in agreement with previous results [9]. This difference can be rationalized in terms of the smaller heterogeneity of genomic DNA-copies (in terms of sequence and fragment-length) and especially of the smaller range of copy number variations compared with the range of variation of mRNA-transcript concentrations. The latter can cover several orders of magnitude whereas the former typically change by less than the factor of ten.

Trivially, the strand direction does not affect the strength of the respective base pairings provided that sequence motifs from both, the s- and the as-strands, are considered in the same direction. In our analyses we therefore pool the probes which are assigned to the same interaction mode independently of their strand direction (d=s, as) assuming that the respective genotypes are properly assigned on both strands.

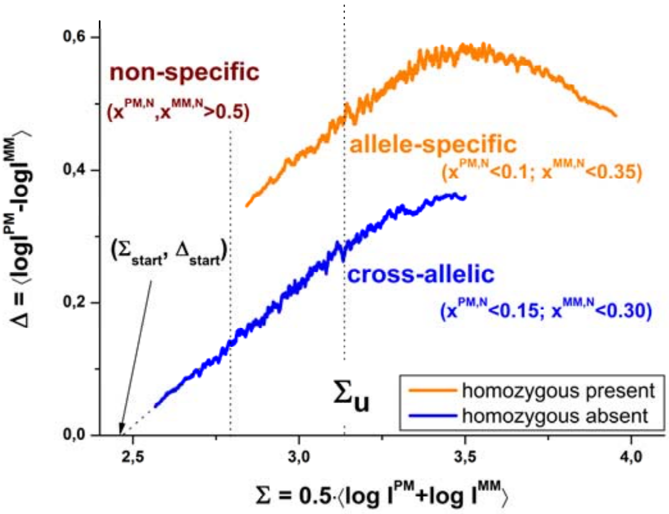

Figure: **Classification of probe-intensities according to their hybridization mode**. So-called hook curves are plotted for homozygous-absent (ha) and -present (hp) probes referring to cross-allelic and allele-specific hybridization modes, respectively. The 'start' coordinates of the hook curve are given by the intersection of the extrapolated ha-hook with the abscissa. The intensity fraction per probe due to non-specific binding depends on the hook coordinates (see Eq. (E1)). The right vertical line refers to $(\Sigma - \Sigma_{start}) > 0.7$. It was used as threshold for probe selection to characterize the interaction modes upon specific (S) and cross-allelic (C) hybridization. Above this threshold, probe intensities are distorted, on the average, by a contribution of non-specific hybridization of less than 20%. The fraction of non-specific binding slightly differs between the PM and MM probes as indicated in the figure.

5. Background correction and saturation effects

The figure (panels a and b) shows triple averaged mean intensities for all 64 standard triples with centre pairings taken from the At-group (WC pairings) and from the Aa-group (self complementary pairings, see also the next section). The data refer either to #mm=0 and 1 mismatches per duplex (At-group) or to #mm=1 and 2 (Aa-group). The mean intensity level decreases with increasing #mm as discussed in the previous section. The different triples of each class give rise to considerable variability of the intensity values. The standard deviation of the whole set of 64 triples of the At-group is SD(logI)=0.041 and 0.045 for #mm=0 and 1, respectively (part a of the figure), but more than twice as large for the mismatched Aa- (SD=0.12; part b of the figure), Ag- (SD=0.13) and Ac-groups (SD=0.09) for #mm=1 (see also Table 1). Hence, mismatched pairings with adjacent WC pairs give rise to considerably larger variation of duplex stability than triples of WC pairs.

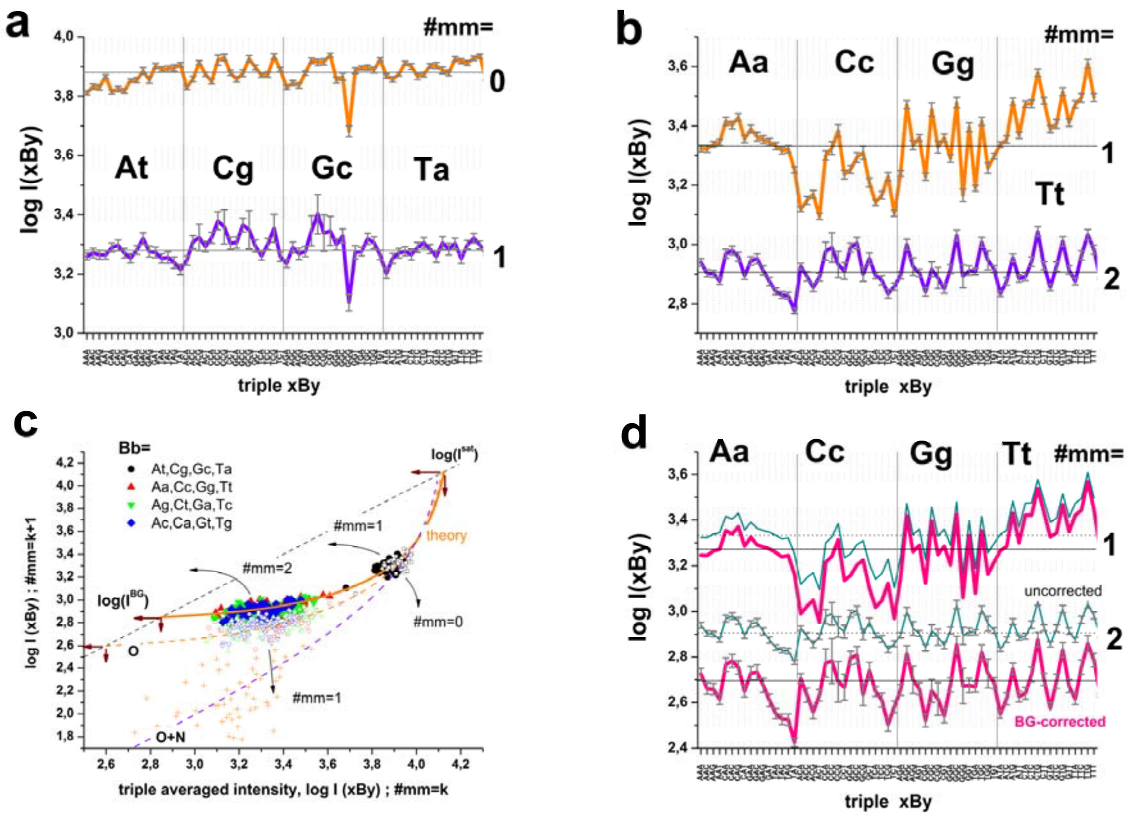

Figure: **Triple-averaged probe intensities and background contribution**. Panel a and b show the 64 triple averaged log-intensities of the perfect match- (At-group) and self complementary mismatch- (Aa-group) pairings. The data refer to different numbers of total mismatches per duplex (#mm, see the figure; the triples are sorted according to their central pairing Bb). These triple averages were correlated for #mm=0-versus-1 and #mm=1-versus-2 in panel c. Here also data for the mismatch-groups Ag and Ac are added. The data do not group in parallel with respect to the diagonal owing to the residual background intensity. Its consideration predicts the grouping of the data along the thick theoretical curve which was calculated using Eq. (E2) with g=11. This curve intersects the diagonal line at the background and saturation intensities, $\log I^{O}$=2.85 and $\log I^{sat}$=4.1, respectively. Correction of the intensities for the optical background (curve “O”) slightly improves the linear correlation between the intensities, especially for #mm=1-versus-2 (open symbols). Consideration of the non-specific background ($\log I^{N}$=2.6) further improves linear correlation, however also inflates variation of the data (see also curve “O+N”). Panel d shows the triple-data of the Aa-group before (thin lines) and after (thick lines) background-correction using Eq. (E3).

In general, one expects the similar base-specific effect independently of the total number of mismatches per duplex. To assess this assumption we correlate the triple averaged log-intensities for #mm=k with that for #mm=k+1, i.e. for duplexes which differ by one mismatched pairing (see part c of the figure). Especially the triple-data of the mismatched groups (Aa, Ag, Ac) do not group in parallel with respect to the diagonal line. This behavior indicates poor correlation (solid symbols, see

also part b of the figure which shows the data for the Aa-group with #mm=1 and 2) in contrast to the data of the At-group data (#mm=0, 1; part a of the figure).

The discussed intensities contain contributions due to the optical and non-specific background (see Eqs. (2) and (4)). Moreover, the intensities saturate at large transcript concentrations and/or binding constants $K_{duplex}(\#mm)$. Let us describe the probe intensities by the hyperbolic function of $K_{duplex}(\#mm)$ [23,57]

$$I(\#mm) \approx \left(\frac{I^{sat} \cdot c \cdot K_{duplex}(\#mm)}{1 + c \cdot K_{duplex}(\#mm)} + I^{BG} \right) \quad . \quad \text{(E2)}$$

I^{sat} denotes the saturation intensity at strong binding, $c \cdot K_{duplex} >> 1$, c is the transcript concentration.

Assuming a factorial increment of the binding constant per mismatch, $K_{duplex}(\#mm+1) = K_{duplex}(\#mm)/g$ (see right axis in Figure 2, panel b), and varying "c $K_{duplex}(0)$" in the limits $0 < c \cdot K_{duplex}(0) < \infty$ we get the theoretical relation between the mean intensities of duplexes which differ by one mismatched pairing (see the curves in panel c of the figure). The theoretical curves intersect the diagonal line (y=x) at low and high intensities at $I=I^{BG}$ and $I=I^{sat}$, respectively, because Eq. (E2) assumes that background and saturation levels are not affected by the number of mismatches. Eq. (E2) predicts significant deviation from the linear relation between the intensities for #mm and #mm+1. The thick curve in panel b of the figure was calculated assuming a residual background intensity of $\log I^{BG} \approx 2.85$. It explains the lack of linear correlation between the experimental triple data for #mm=0-versus-1 and especially of #mm=1-versus-2.

The used background refers to the optical and non-specific contributions according to Eq. (4). To estimate the optical background we simply select 1% smallest intensity probes of the array, calculate their log-intensity average ($\log I^{O}=2.39$), and correct the intensities for this contribution, $I^{corrO}= I - I^{O}$ (see open symbols in panel c of the figure). The dashed curve labeled with "O" refers to these data containing a contribution due to non-specific background intensity of about $\log I^{N} \approx 2.65$. Intensity data which are corrected for both contributions, $I^{corrO+N}= I - I^{BG}$, are shown by the small crosses. The respective theoretical curve labeled "O+N" runs parallel with the diagonal line at decreasing intensities.

The total background correction markedly inflates the variability of the data at small intensities. This effect is well known from microarray analyses as the consequence of diverging log-transformed data at vanishing argument. To avoid this trend it is common practice to confine the corrected data to a lower limit, for example by adding a small constant value to the corrected intensities. We also apply this modification using ($\log I^{N} - o$) with o= 0.6 instead of $\log I^{N}$.

So far we estimated the mean optical and non-specific background levels which apply to all probes of the chip. The background contribution due to non-specific hybridization is governed by the binding reaction of non-specific transcripts (see Eq. (1)). It consequently depends on the probe sequence and thus it is specific for each probe. We previously showed that non-specific hybridization is basically characterized by Watson-Crick pairing [18]. Final background correction of the triple averaged intensities was therefore applied in a sequence specific fashion using

$$I^{corr}(xBy) = I(xBy) - I^{O} - I^{N} \cdot 10^{-o+Y_{At}(xBy)} \quad \text{(E3)}$$

where $Y_{At}(xBy)$ is the sensitivity of the respective triple of the At-group (see Eq. (8)).

This correction progressively reduces the mean intensity level for #mm=1 and #mm=2 (see Figure 2, part b and the figure above, part d). The triple-specific effect is almost negligible for #mm≤1 but it affects the results for #mm=2.
